# A Progressive Loss of phosphoSer138-Profilin Aligns with Symptomatic Course in the R6/2 Mouse Model of Huntington’s Disease: Possible Sex-Dependent Signaling

**DOI:** 10.1007/s10571-020-00984-2

**Published:** 2020-10-27

**Authors:** Akanksha Baharani, Zelan Wei, William J. Roesler, Darrell D. Mousseau

**Affiliations:** 1 Department of Biochemistry, Microbiology, and Immunology, University of Saskatchewan, GA20, Health Sciences Building, 107 Wiggins Rd, Saskatoon, SK S7N 5E5 Canada; 2 Cell Signalling Laboratory, Department of Psychiatry, University of Saskatchewan, GB41 Health Science Building, 107 Wiggins Road, Saskatoon, SK S7N 5E5 Canada

**Keywords:** Kinome analysis, Huntington disease, Cytoskeleton, Neurodegeneration, Developmental

## Abstract

The R6/2 transgenic mouse model of Huntington’s disease (HD) carries several copies of exon1 of the *huntingtin* gene that contains a highly pathogenic 120 CAG-repeat expansion. We used kinome analysis to screen for kinase activity patterns in neural tissues from wildtype (WT) and R6/2 mice at a pre-symptomatic (e.g., embryonic) and symptomatic (e.g., between 3 and 10 weeks postnatal) time points. We identified changes in several signaling cascades, for example, the Akt/FoxO3/CDK2, mTOR/ULK1, and RAF/MEK/CREB pathways. We also identified the Rho-Rac GTPase cascade that contributes to cytoskeleton organization through modulation of the actin-binding proteins, cofilin and profilin. Immunoblotting revealed higher levels of phosphoSer138-profilin in embryonic R6/2 mouse samples (cf. WT mice) that diminish progressively and significantly over the postnatal, symptomatic course of the disease. We detected sex- and genotype-dependent patterns in the phosphorylation of actin-regulators such a ROCK2, PAK, LIMK1, cofilin, and SSH1L, yet none of these aligned consistently with the changing levels of phosphoSer138-profilin. This could be reflecting an imbalance in the sequential influences these regulators are known to exert on actin signaling. The translational potential of these observations was inferred from preliminary observations of changes in LIMK-cofilin signaling and loss of neurite integrity in neural stem cells derived from an HD patient (versus a healthy control). Our observations suggest that a pre-symptomatic, neurodevelopmental onset of change in the phosphorylation of Ser138-profilin, potentially downstream of distinct signaling changes in male and female mice, could be contributing to cytoskeletal phenotypes in the R6/2 mouse model of HD pathology.

**Electronic supplementary material:**

The online version of this article (10.1007/s10571-020-00984-2) contains supplementary material, which is available to authorized users.

## Introduction

Huntington’s disease (HD) is an autosomal-dominant, progressive neurodegenerative disorder that affects 5–7 per 100,000 people (Wynford-Thomas and Robertson 2017). The genetic defect involves a CAG (trinucleotide coding for glutamine/Gln/Q) repeat expansion within exon 1 of the *huntingtin* gene (*HTT*). Above the pathological threshold (≥ CAG39; Q39), there is a strong correlation between the number of repeats and the age of onset and/or severity of clinical manifestation (Ross and Tabrizi 2011).

The wildtype (WT) HTT protein functions in a number of processes including energy metabolism, synaptic function, protein transport, transcription, survival, autophagy, and cytoskeletal dynamics [reviewed in (Saudou and Humbert 2016)]. A reduction in WT HTT has been implicated in axonal trafficking defects (Trushina et al. 2004), while the deletion of WT HTT significantly attenuates regeneration, hence implicating it in neural plasticity after injury (Poplawski et al. 2020; Belin et al. 2015). Consistent with this, the mutant huntingtin protein (mHTT) can impact proteins involved in a diverse range of biological processes (Culver et al. 2012; Hosp et al. 2017). The mHTT targets primarily medium spiny neurons in the striatum, a structure enriched in dopamine (DA) neurotransmission (Roze et al. 2011), which increases in early stage of the clinical course of HD (leading to hyperkinetic movements) and decreases as pathology progresses, including in mouse models of HD (Cepeda et al. 2014). The progressive disruption in striatal DA transmission and any synaptic plasticity defect might rely, in part, on interference of the critical interaction between DA receptors, components of the actin cytoskeleton (especially Filamin-A, which participates in the anchoring of membrane proteins to the actin cytoskeleton), and downstream DA signalling molecules (Lin et al. 2001); this might occur during development and affect synaptogenesis (McCarthy et al. 2011; Zhang et al. 2010).

The potential for sex-dependent differences in HD progression and severity of phenotype is unclear. For example, women can present with a slightly more severe phenotype and a faster progression of HD than men (Zielonka et al. 2013), although contrasting reports also suggest that symptom onset is later (Roos et al. 1991) and that disease progression is milder (Roos et al. 1991; Chen et al. 2009) in women. Part of the substantial loss of striatal DA receptors and severity of phenotype in symptomatic male (versus female) transgenic HD rats (Q51) has been attributed to lower levels of the neuroprotective sex hormone 17β-estradiol in the male rat (Bode et al. 2008). A role for estrogen in clinical HD is supported by the demonstration that the phytoestrogen, genistein, promotes the breakdown of mHTT in HD fibroblasts (Pierzynowska et al. 2019) and by a much earlier demonstration that premarin could improve motor symptoms, but in less than 30% of patients (Koller et al. 1982). Clearly, examination of a role for estrogen in the context HD is warranted.

We undertook a preliminary comparison of neural stem cell cultures and observed dysregulation of the cytoarchitecture (e.g. a loss of neurite outgrowth) in HD (Q45) cultures (cf. healthy control). Western blotting confirmed changes in molecular signatures implicated in actin signalling, such as changes in LIMK1, cofilin, and SSH1L (all components of the Rho-Rac signalling pathway). In order to identify changes in signaling pathways during the symptomatic course of HD, we chose to use the R6/2 mouse model. This transgenic mouse carries copies of a fragment of exon 1 of the human *huntingtin* gene containing a Q120 repeat expansion that is sufficient to trigger a progressive behavioral and neurological HD-like phenotype that manifests by 4–6 weeks of age (Mangiarini et al. 1996). We used kinome analysis (Berard et al. 2018) to screen for potential phosphorylation events (Jalal et al. 2009) and identified several affected signaling cascades, including the Rho-Rac GTPase cascade that has been associated with cytoskeletal phenotypes in various models of HD (Puigdellivol et al. 2015; Tourette et al. 2014; Tousley et al. 2019). A role for this cascade was corroborated by evaluating the phosphorylation status of key signaling proteins such as ROCK, LIMK1, SSH1L, cofilin, and profilin in R6/2 mouse tissues. Our observations strongly suggest distinct signaling changes in male and female mice, and as importantly, indicate an onset of signalling defect centered on the HTT- and actin-binding regulator profilin (Shao et al. 2008; Angeli et al. 2010) at embryonic stages, thus corroborating the suggestion that HD progression might have a neurodevelopmental origin (Wiatr et al. 2018).

## Materials and Methods

### Neural Stem Cell Cultures

Neural stem cells (NSCs) were derived from iPSCs (induced pluripotent stem cells) obtained from a female HD (Q45) donor (ax0021) and from an age-matched female healthy control (HC) donor (ax0016) (Axol Bioscience, Cambridge, UK). Culture dishes were coated with Axol Sure Bond coating solution (ax0041) prepared in PBS (without calcium or magnesium; D-PBS) overnight at 37 °C. iPSCs were seeded at a density of 10,000–50,000 cells/cm^2^ in Axol Neural Maintenance Media (ax0031) supplemented with the Axol Sure Boost serum (ax0045) for 2 h and then cultured for 48 h in Neural Maintenance Medium supplemented with the Axol Sure Growth serum (ax0047). Thereafter, cells were cultured in the Neural Maintenance Medium alone. For passaging and harvesting of NSCs, the cultures were rinsed with PBS and detached using the Axol Neural Unlock solution (ax0044). An Olympus CKX41 light microscope was used for assessing neurite outgrowth, cell number, and soma size (quantitation was performed using Neurolucida360 software: MBF Bioscience, Williston, VT).

### Animal Tissue Harvest

All animal procedures were performed in accordance with Canadian Council on Animal Care guidelines and were approved by the University of Saskatchewan's Animal Research Ethics Board. Animals had access to food and water ad libitum, and were housed under constant temperature (± 22 °C) and humidity (± 45%) with a 12:12 h light/dark cycle. Age-matched breeding pairs of R6/2 transgenic mice (#6494) were purchased from the Jackson Laboratory (Farmington, CT). Tissues were harvested at embryonic day 14 (E14), when the striatum begins to develop (Voorn et al. 1988), as well as at a postnatal pre-symptomatic stage (at 3 weeks of age), a stage when striatal mHTT immunoreactivity is first detectable (at 5 weeks), and a stage when overt symptoms—such as brain and body weight loss, and a visible motor phenotype—are evident (at 10 weeks) (Davies et al. 1997) (Supplementary Fig. 1). Given the lack of a defined striatum at E14, whole brain were used for analyses at this time-point. The striatum was used for analyses at postnatal stages, e.g. 3-week (3w), 5w, and 10w. In all cases, mice were euthanized by cervical dislocation.

### Genotyping and PCR

Embryos (skull tissues) and pups (tail snips) were genotyped for the *HTT* transgene and sexed using *SRY* (sex-determining region Y protein). DNA was extracted (Qiagen kit: # 60506) and PCR amplification was carried out using Phusion® DNA polymerase in combination with the *HTT* primer pair: (forward) 5′-CCG CTC AGG TTC TGC TTT TA-3′ and (reverse) 5′-TGG AAG GAC TTG AGG GAC TC-3′; or the *SRY* primer pair: (forward) 5′-TTG TCT AGA GAG CAT GGA GGG CCA TGT CAA-3′, and (reverse) 5′-CCA CTC TGT GAC ACT TTA GCC CTC CGA-3′. Primers were purchased from Invitrogen Life Technologies (New York, NY).

### Peptide Arrays and Kinome Analyses

DAPPLE 2 (https://saphire.usask.ca/saphire/dapple/) was used to design the peptide arrays (Trost et al. 2013a). The customized peptide microarray (JPT Peptide Technologies GmbH, Germany) contained 1268 peptides (corresponding phosphosites are listed in Supplementary Table 1) designed to cover key signaling pathways (Jalal et al. 2009). Only those murine proteins (corresponding peptides) that have a human homolog were selected to populate the microarray. The selection was accomplished using web-based online databases such as Phosphosite plus (Hornbeck et al. 2012). There were five biological replicates (5 separate arrays) performed, with nine technical replicates per array. The resulting 45 intensity values for every peptide per biological sample were normalized using ‘variance stabilization normalization’ transformation and the difference in the fold-change and *P*-values were calculated using PIIKA-2 (Platform for Intelligent, Integrated Kinome Analysis), as described in detail elsewhere (Jalal et al. 2009; Trost et al. 2013b) (Supplementary Table 2). The software (InnateDB) considers both fold-changes and *P*-values to define significantly dysregulated pathways (Breuer et al. 2013) and draws upon information from several major academic databases, including KEGG, REACTOME, and INOH, to generate a list of candidate pathways. The pathways analysis revealed upregulated as well as downregulated pathways [provided in Supplementary Tables 3 and 4, respectively].

**Table 1:** The list of hyper- and hypo-phosphorylated peptides in both sexes across four developmental time points

Time points	Hyper-/hypo-phosphorylated	Female: peptides	Male: peptides	Common peptides
E14	Hyper-	416	292	119
	Hypo-	392	344	105
3w	Hyper-	462	390	162
	Hypo-	362	228	75
5w	Hyper-	392	401	66
	Hypo-	335	379	58
10w	Hyper-	373	334	118
	Hypo-	291	339	75

The developmental time points at which the R6/2 murine tissue were harvested are listed in the first column. The next three columns list the number of peptides that were significantly (*P* < 0.2) hyper- and hypo-phosphorylated based on the kinome analysis in female and male mice. The last column lists the number of hyper- or hypophosphorylated peptides that were in common in both sexes

### Western Blot Analysis

Tissues were sonicated in RIPA buffer on ice with five 40 mA pulses (3 s each, separated by a 10 s pause), centrifuged at 12,000×*g* (4 °C, 30 min), and supernatants were heat-denatured. Samples (20 µg protein) were resolved by SDS-PAGE and transferred to nitrocellulose membranes, which were blocked and probed with primary antibodies. Detection relied on Image Studio™ Lite software (LI-COR) and densitometry was normalized to α-/β-tubulin levels.

### Antibodies for WB Analyses

Antibodies directed against cofilin (cat #: 3311), phosphoSer3-cofilin (3318), profilin1 (3237), ROCK2 (8236), PAK (2604), phosphoSer473-AKT1 (9018S), AKT1 (2938S), phosphoFoxO1/3a/4 (2599), FoxO1 (2880S), FoxO3a (12829), FoxO4 (9472S), and the MAPKAPK-2 Kit (9329; includes phosphoThr222 and -Thr234) were purchased from Cell Signaling Technologies (Danvers, MA). Antibodies recognizing LIMK1 (ab38508), phosphoThr508-LIMK1 (ab95186), phosphoThr423-PAK (ab2477), phosphoSer138-profilin1 (ab215752), phosphoSer1366-ROCK2 (ab228008), phosphoT160-CDK2 (ab194868), CDK2 (ab32147), and α-Tubulin (ab4074) were purchased from Abcam (Cambridge, MA). Slingshot (SSH1L) and phospho-Slingshot antibodies (SK6410) were purchased from Cedarlane (Burlington, ON). The anti-β-tubulin antibody (T8328) was purchased from Sigma-Aldrich (Oakville, ON). Secondary antibodies including IR Dye-680RD IgG (926-68071), IR Dye-800CW IgG (926-32211), and IR Dye-800CW IgG, (926-32210) were purchased from LI-COR Biosciences (Lincoln, NE).

### Statistical Analysis

A peptide was selected from PIIKA2 output for further analysis if its *P*-value was < 0.2 along with a fold-change (FC) >  ± 1 (Goel et al. 2018; Maattanen et al. 2013). The *P*-value of 0.2 was chosen as it is known that if the threshold were to be too conservative, then the likelihood of false negatives would increase, and if too relaxed, then the analysis might provide false positives. Further, given that a cellular phenotype is often the reflection of changes in the expression patterns of groups of signaling molecules with common biological functions, identifying a change in a group of these molecules is more biologically meaningful than a change in a single molecule. As importantly, it has been noted that 50–70% of the information from peptide arrays can be lost due to technical reasons during data normalization (Scholma et al. 2016). The cut-off threshold used for InnateDB pathway analysis was more stringent (*P* < 0.05 and FC >  ± 1.5), with *P*-values being generated using the hypergeometric distribution test that confirms—prior to correction for multiple testing—whether a pathway is statistically more over-represented in the uploaded dataset than expected by chance. *P*-values are automatically corrected using the Benjamini and Hochberg or by a conservative Bonferroni correction (Breuer et al. 2013). Our kinome analyses relied on 5 males and 5 females per genotype per test time-point. The priority of kinome analysis is to identify targets that can be validated by an independent approach, for example, Western blotting.

Western blot bands were quantified using Image Studio Lite (LI-COR Biosciences) and the intensities were normalized using housekeeping control (α/β-tubulin). The phospho-proteins were expressed relative to total protein and the corresponding ratios were used for statistical analyses based on two-way analysis of variance (ANOVA) and post hoc Tukey's multiple comparison test (GraphPad v7, PRISM). Morphological features of NSCs were estimated using six separate fields from several HC and HD cultures, and averages were compared using the Student *t*-test. Statistical significance was set at *P* < 0.05. All data are expressed as mean ± standard error of the mean. Our Western blotting relied on 3 males and 3 females per genotype per test time-point.

## Results

### Neurite Retraction in Patient-Derived HD Neuronal Cells

**Fig. 1:**
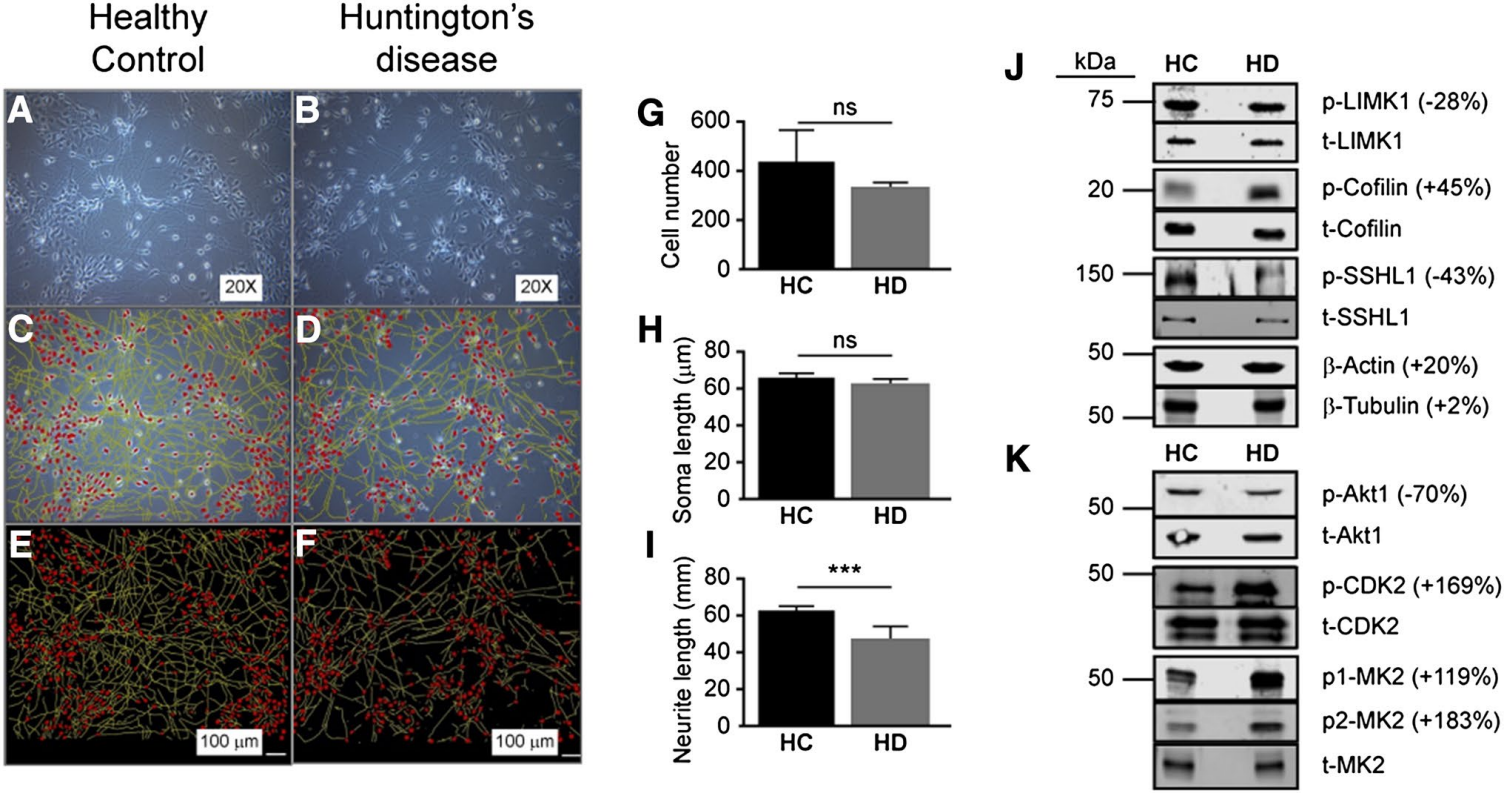
Examination of neural stem cell (NSC) cultures: representative phase-contrast images of **a** healthy control (HC) and **b** Huntington’s disease (HD; Q45) NSCs cultured to approximately 80% confluence. **c**, **d** Reconstruction of the neurite extensions were generated using Neurolucida 360 and the representative overlay indicating the soma and neurites are depicted. **e**, **f** Tracings used for quantification of neurite lengths. **g** Average cell number in HD and HC NSCs. **h** Average size of cells in HD and HC NSC cultures. **i** Average neurite length of HC and HD NSCs. **j** Representative Western blots of selected proteins implicated in cytoskeletal integrity, e.g. LIMK1, Cofilin, Slingshot (SSH1L), β-actin, and β-tubulin. **k** Representative Western blots of other candidate signaling targets, e.g., Akt, CDK2, and MAPKAPK2 (MK2). p1-MK2 = phosphoThr222; p2-MK2 = phosphoThr234. Numbers in parentheses indicate the percentage change in densitometry of bands in HD NSC extracts relative to HC NSC extracts. Bar graph shows mean ± standard deviation, ***P < 0.005, n = 6 replicates

Comparison of NSC cultures derived from a healthy control (HC) (Fig. 1a, c, e) and an HD patient and from (Fig. 1b, d, f) did not reveal any significant loss of cell number (Fig. 1g) or change in shape of the cell (Fig. 1h). However, there was a 25% decrease in neurite length in the HD NSCs (*P* < 0.05) (Fig. 1i). Western blotting for selected proteins implicated in actin organization and cytoskeletal integrity revealed less phosphorylation of LIMK1 in the HD NSC lysate, but more phosphorylation of the LIMK1 substrate, cofilin; the latter might be reflecting the lower levels of phosphorylation (and, hence, inactivation) of the cofilin phosphatase, SSH1L (Fig. 1j). Levels of actin were higher in the HD NSC lysate, whereas those of β-tubulin remained unaltered. Preliminary Western blotting of the NSC extracts also revealed changes in the phosphorylation of the pro-survival kinase Akt (− 70%), the cell cycle regulator CDK2 (+ 169%), and the stress-activated kinase involved in cytoskeletal organization, cell cycle, and chromatin remodeling, MAPKAPK2 (+ 119–183%) (Fig. 1k) (discussed below).

These data suggest that the loss of communication between cells in HD might rely primarily on a loss of axonal integrity and synaptic connectivity, and implicate a potential influence by the LIMK/SSH1L/cofilin pathway. However, the interpretation of these data is hindered by the fact that the iPSCs available from the commercial source at the time were limited to a single HC female donor and a single sex-/age-matched HD donor (leaving us with a biological replicate of ‘1’). This precluded any possibility of concluding whether the observed changes were due to the sex of the donor, the diagnosis of HD, an interaction between sex and diagnosis, or even variation within the cultures (given that they were non-isogenic). Yet we feel that the observations, even if based on a single biological replicate, clearly indicated a cytoskeletal defect and implicated the LIMK/SSH1L/cofilin pathway, and thus provided justification for in vivo studies. To this end and to explore whether sex might exert influence, we screened protein kinase activities in the R6/2 mouse model of HD (and WT mice) using kinome analysis. Our experiments included both male and female mice.

### The Phospho-protein Profiles in R6/2 Mice Suggest Distinct Sex-Dependent Influences on Signaling

**Fig. 2:**
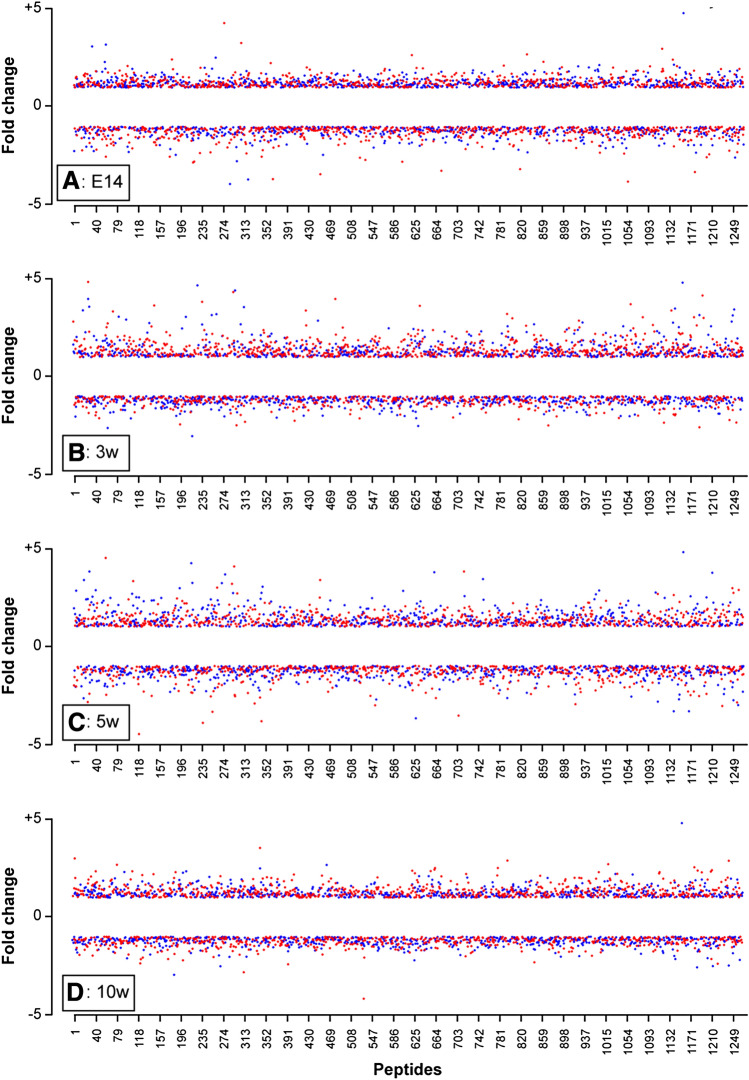
Fold-changes in individual peptide phosphorylation states identified by kinome analysis uncovers sex differences in R6/2 mice at time-points across the life-span: **a** at E14, **b** at 3w, **c** at 5w, and **d** at 10w. Peptide numerical codes are listed along the x-axis and the corresponding fold-change values on the y-axis. The females are represented by red dots and males by blue dots. The positive values indicate hyper-phosphorylated peptides and negative values represent hypo-phosphorylated peptides. The corresponding peptides and their relative changes are summarized in Supplementary Tables 1 and 2, respectively

Kinome analysis identified peptides that were significantly hyper- and hypo-phosphorylated at a pre-symptomatic stage (E14) (Fig. 2a) as well as across all three postnatal, symptomatic time points (Fig. 2b–d). The analysis revealed peptides that were similarly hyper/hypo-phosphorylated in both sexes and others that were preferentially hyper/hypophosphorylated by sex (Table 1).

The kinome analysis data (Supplementary Table 2) were uploaded onto InnateDB along with their respective *P*-values and fold-changes (FC); this generated a list of pathways that were up-/down-regulated across the time course in these mice (provided in Supplementary Tables 3 and 4). The top three most significantly upregulated pathways were ‘Caspase mediated cleavage of cytoskeletal proteins’ (*P* = 7.17E−04; REACTOME), ‘Lysosomes’ (*P* = 8.91E−04; KEGG), and ‘Peptide ligand binding receptors’ (*P* = 0.004190495; REACTOME) (Supplementary Fig. 2). The top three most significantly downregulated pathways were ‘Degradation of DVL’ (*P* = 3.53E−04; REACTOME), ‘Beta-catenin independent WNT signaling’ (*P* = 0.001525682; REACTOME), and ‘Degradation of GLI1 by proteasome’ (*P* = 0.001658169; REACTOME) (Supplementary Fig. 3). Other pathways identified were [upregulated] ‘Depolymerisation of the nuclear lamina’, ‘disinhibition of SNARE formation’, and ‘metabolism of steroid hormones and vitamin D’ and [downregulated] ‘Trafficking of AMPA recycling’, ‘Recycling of L1′, ‘Transmission across chemical synapses’. Although the RhoRac GTPase pathway was not specifically represented in the InnateDB analysis performed, we manually annotated members of the pathway (Supplementary Fig. 4) and identified several changes in phosphorylation of proteins in the kinome analysis. The major components of the ROCK (Rho-associated protein kinase) and PAK (p21-activated kinase) cascades (Supplementary Fig. 4) involve serine/threonine protein kinases (and counterbalancing phosphatases), with primary regulatory effects on the actin cytoskeleton and ultimate phenotypic effects centered on neuronal growth and synaptic plasticity (Zhao and Manser 2012; Julian and Olson 2014). Other preliminary examinations of signaling pathways are included in Supplementary Figs. 5–8. These pathways are included for sake of information and confirm our preliminary Western blotting of the NSC extracts, which also revealed changes in the phosphorylation of the pro-survival kinase Akt, the cell cycle regulator CDK2, and the stress-activated kinase involved in cytoskeletal organization, cell cycle, and chromatin remodeling, MAPKAPK2 (Fig. 1k). These signaling pathways were not explored further in this report.

**Fig. 3:**
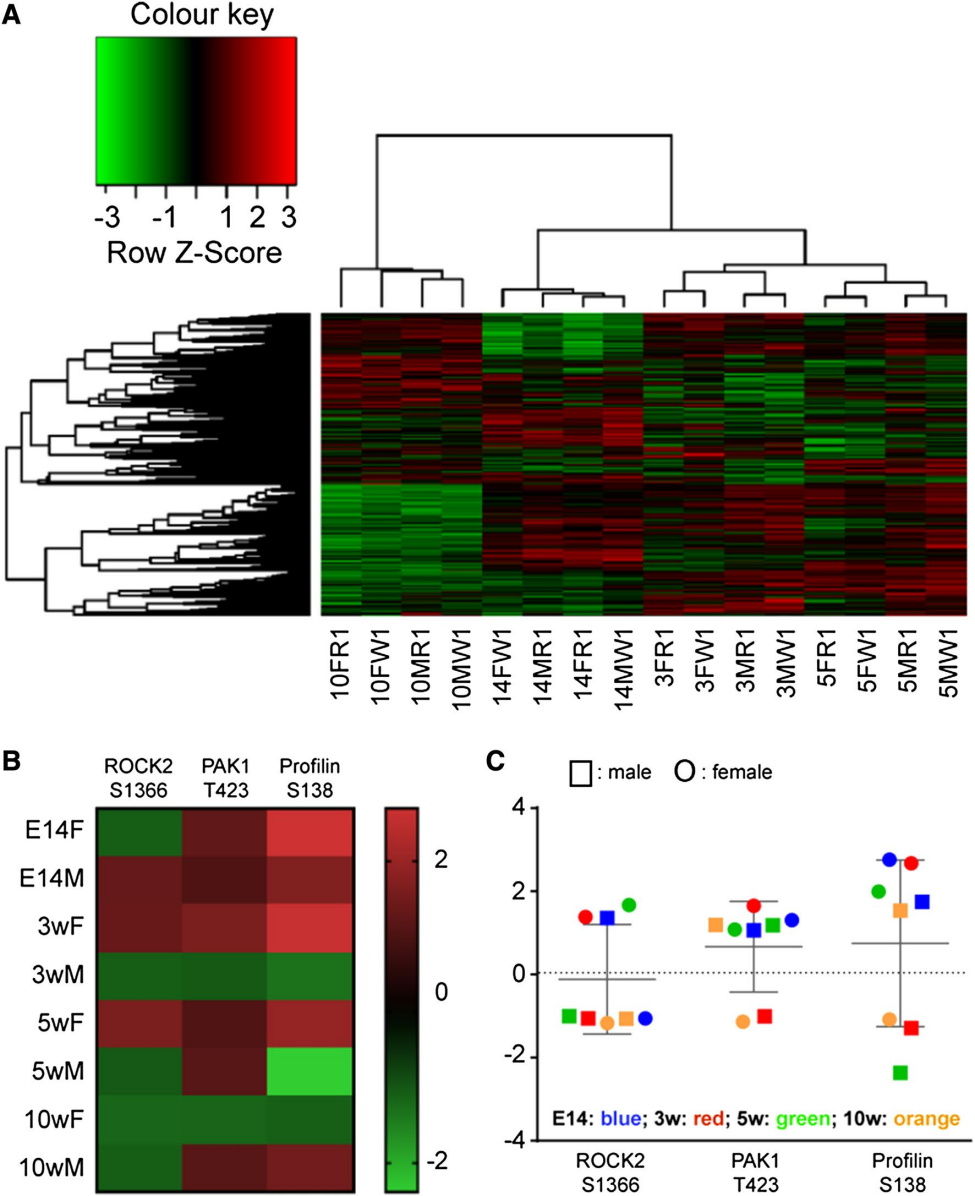
Changes in phosphorylated peptides identified in the kinome analysis of the R6/2 mice: **a** Cluster analysis of kinome data sets of neural tissue samples of R6/2 mice (relative to WT mice). Kinome data sets were subjected to hierarchical clustering analysis using PIIKA-2. The age of the animal is indicated under the heatmap where number represents the time point (E14, 3w, 5w, and 10w) followed by sex (F/M) and the genotype, e.g. WT (W) and R6/2 (R). Each column depicts the kinome activity at that time point. Green represents hypo-phosphorylated peptides and red represents hyper-phosphorylated peptides. **b** Fold-change heatmap for the three indicate phosphopeptides across four time-points, e.g. E14, 3w, 5w, and 10w, in both sexes. The names of the peptides and the phosphosite are indicated at the top of each column. The color key represents positive values in red and negative values in green. **c** Scatterplot of fold**-**changes based on sex. Males are represented as squares and females as circles, with a different color assigned to each time-point, as indicated in the panel

Using our peptide array dataset (Fig. 3a), we extracted the data relating to three of the proteins in the ROCK/PAK cascades (see Supplementary Fig. 4). The respective heatmaps (Fig. 3b) and fold-change values obtained in R6/2 tissues and in control tissues (Fig. 3c) for phosphoSer1366-ROCK2, phosphoThr423-PAK1, and phosphoSer138-profilin highlight sex differences at the various stages. For example, at the E14 stage, Ser1366-ROCK2 is *hyper*phosphorylated in males and *hypo*phosphorylated in females, while Thr423-PAK1 and Ser138-profilin are both *hyper*phosphorylated regardless of sex. In contrast, at the 10w time point, the pattern is completely reversed with Ser1366-ROCK2 being *hypo*phosphorylated (regardless of sex), while Thr423-PAK1 and Ser138-profilin are both *hyper*phosphorylated in males and *hypo*phosphorylated in females (Fig. 3c).

### Validation of Kinome Analysis Data by Western Blot Analysis

**Fig. 4:**
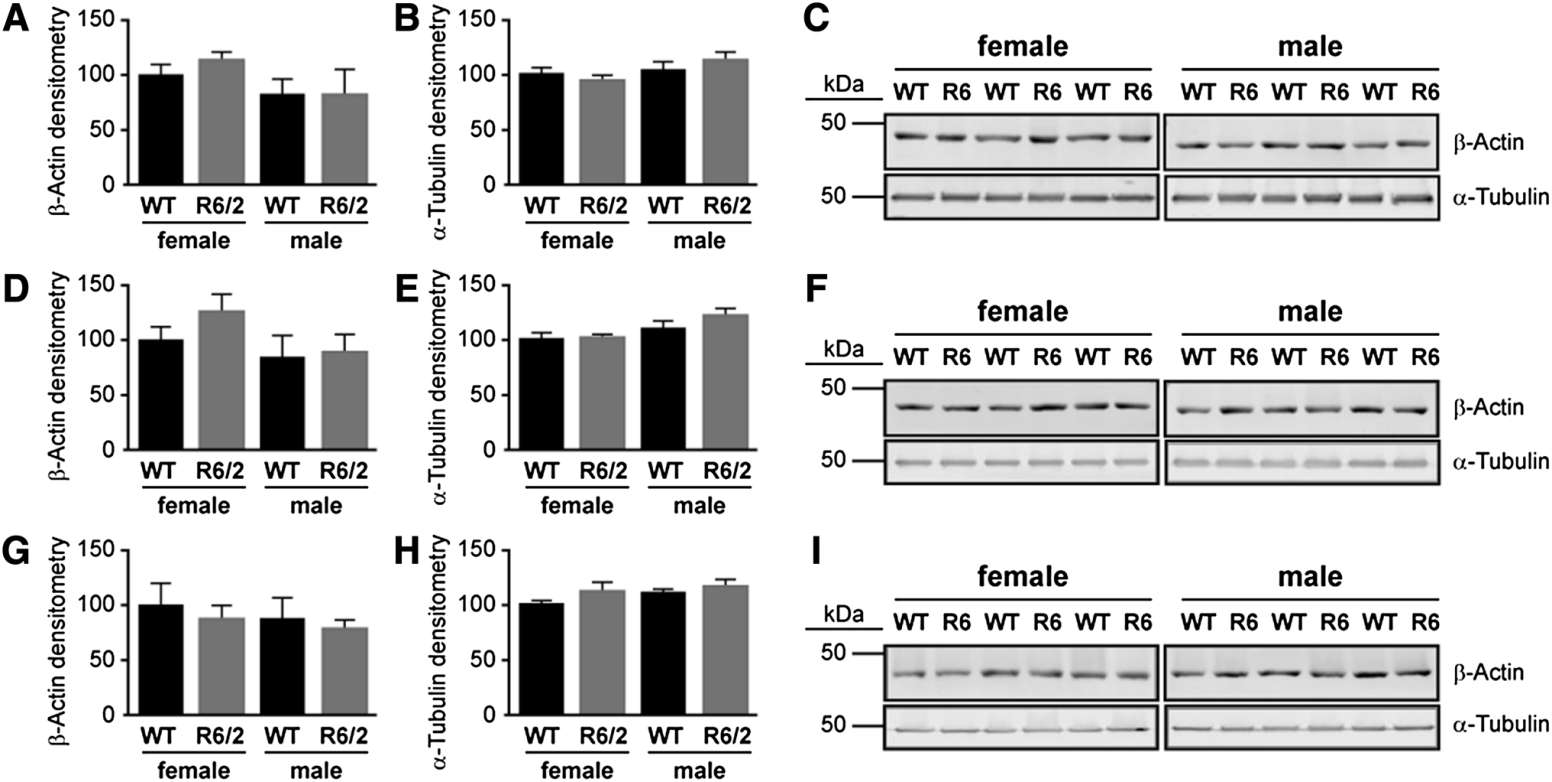
Levels of β-actin and α-tubulin at postnatal stages in WT and R6/2 mice. The time-points were chosen as they represent different stages of disease progression in the R6/2 mouse: e.g. **a**–**c** 3 week old (3w) = preclinical, **d**–**f** 5w = emergence of motor abnormalities; and **g**–**i** 10w = overt pathology. The graphs (left) represent the average expression of β-actin and α-tubulin quantified by densitometry of the corresponding Western blots from striatal samples (right). The data (n = 3) are expressed as % relative to female WT

We investigated the phosphorylation levels of selected proteins in the Rho-Rac pathway at time-points relevant to key stages of HD progression in the R6/2 mouse model, namely developmental (E14), pre-symptomatic (3w), early disease (5w), and late-stage disease (10w). The levels of β-actin tended to be more variable than those of α-tubulin between the sexes and across genotypes (Fig. 4a–i). Consequently, levels of α-tubulin were used to monitor protein loading in the Western blotting experiments.

**Fig. 5:**
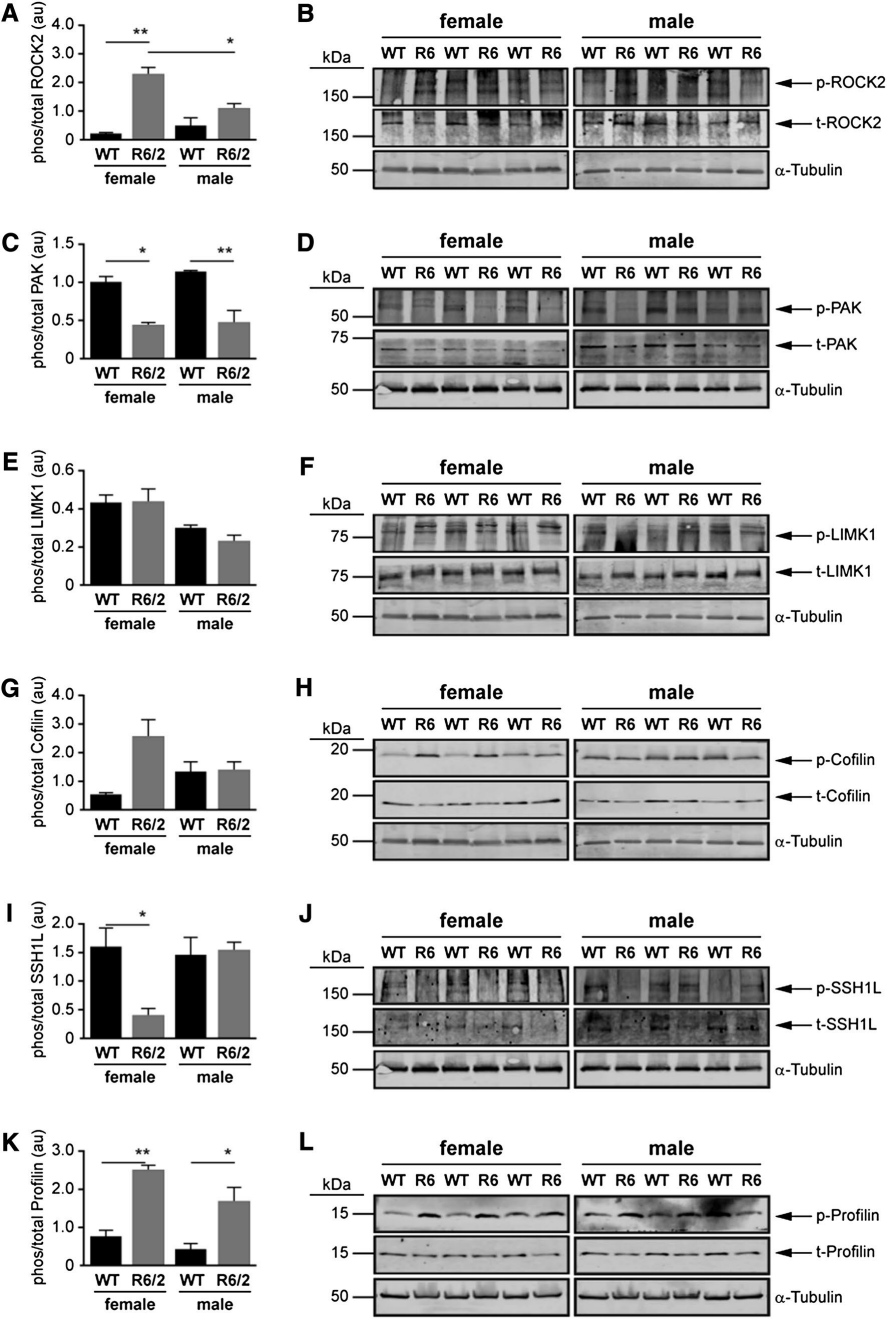
Western blots of the proteins involved in Rho-Rac signaling at E14 in wild type (WT) and R6/2 mice. Densitometry was used to quantify the ratio of phosphorylated to total protein expression of **a**, **b** ROCK2, **c**, **d** PAK, **e**, **f** LIMK1, **g**, **h** Cofilin, **i**, **j** SSH1L, **k**, **l** Profilin. Each value was initially normalized to expression of α-tubulin in the corresponding lane. Note that the same α-Tubulin blot might appear in more than one panel because of re-probing for multiple targets with non-overlapping molecular weights on a given blot. The data are presented as mean ± sem (n = 3). *P < 0.05; **P < 0.01, between indicated groups

At the E14 time-point, the phosphorylation of ROCK2 was significantly elevated in female R6/2 mice compared to female WT mice (*P* < 0.0001) and male R6/2 mice (*P* < 0.05) (Fig. 5a, b). The phosphorylation of PAK was lower in both sexes in R6/2 mice compared to WT mice (Female: *P* < 0.05; Male: *P* < 0.001) (Fig. 5c, d), while the phosphorylation of LIMK1 and cofilin were not affected by sex or genotype (Fig. 5e–h). The phosphorylation of SSH1L was lower in female (*P* < 0.05)—but not male—R6/2 mice (Fig. 5i, j). The phosphorylation of profilin was substantially elevated in both sexes in R6/2 mice compared to WT mice (female: *P* < 0.001; male: *P* < 0.05) (Fig. 5k, l).

**Fig. 6:**
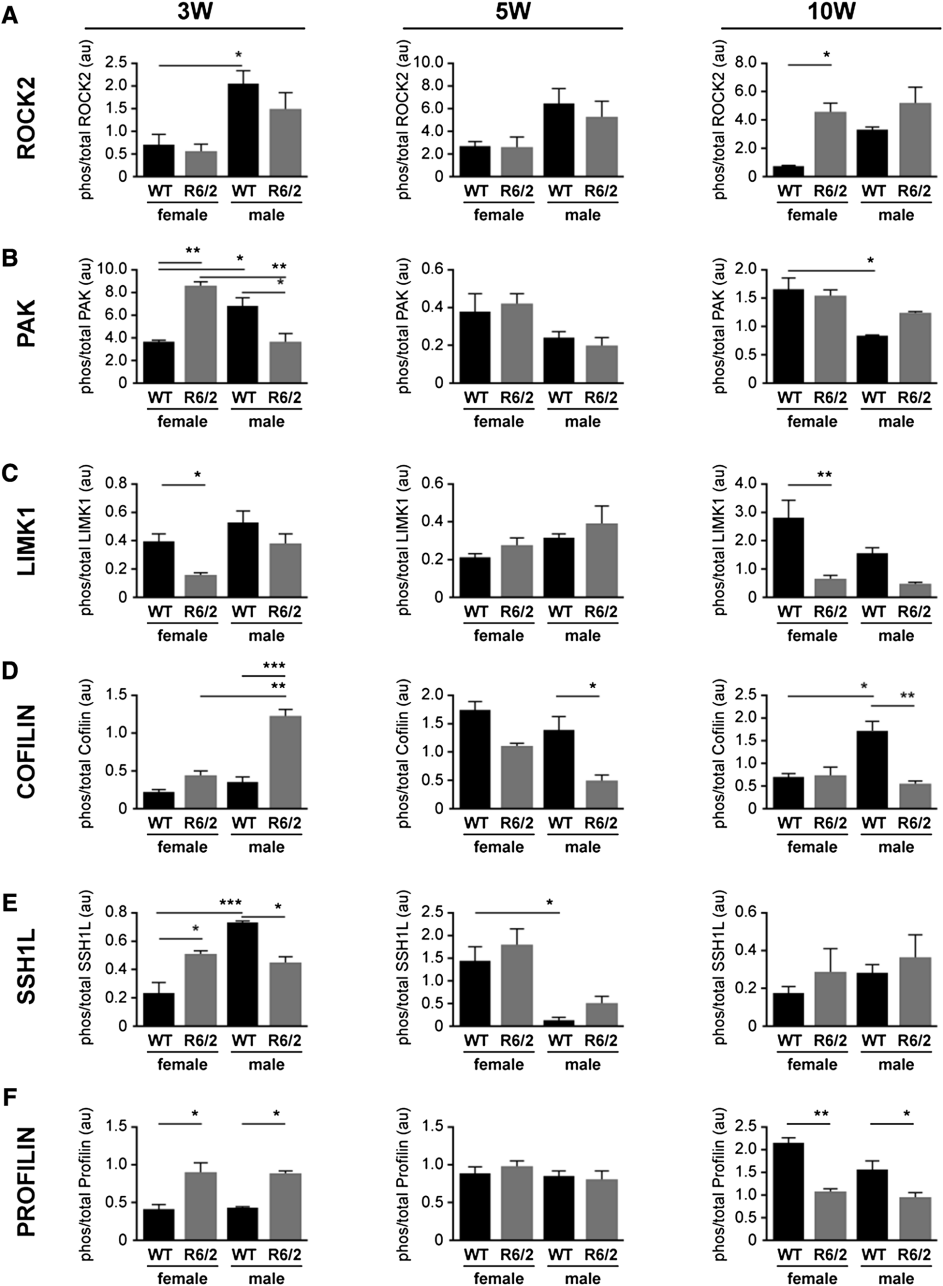
Densitometric analysis of the proteins involved in Rho-Rac signaling at postnatal time-points in wild type (WT) and R6/2 mice. The three time-points represent different stages of disease progression in the R6/2 mouse: e.g. 3 week old (3w) = preclinical; 5w = emergence of motor abnormalities; and 10w = overt pathology. Densitometry was used to quantify the ratio of phosphorylated to total protein **a** ROCK2, **b** PAK, **c** LIMK1, **d** Cofilin, **e** SSH1L, **f** Profilin. Each value was initially normalized to expression of α-Tubulin in the corresponding sample. The data are presented as mean ± sem (n = 3). *P < 0.05; **P < 0.01; ***P < 0.001, between indicated groups

There was significant variability in the phosphorylation of the proteins examined at the 3w time-point, when a mouse is considered juvenile and the brain is thought to be still maturing. For example, the phosphorylation of ROCK2 was higher in WT males than in WT females (*P* < 0.05), but was unaffected by genotype (Fig. 6a). The level of phosphorylation of PAK was higher (or lower) depending on the sex and genotype (Fig. 6). The phosphorylation of LIMK1 was lower in the female R6/2 mouse compared to the WT females (*P* < 0.05) (Fig. 6c), while the phosphorylation of cofilin was higher in the male R6/2 mouse (cf. male WT and female R6/2 mice) (*P* < 0.0001) (Fig. 6d). The phosphorylation of SSH1L was similar to that of PAK in that in that it was higher (or lower) depending on the sex and genotype (Fig. 6e).

In contrast, only sporadic differences were observed at the 5w (e.g. emergence of motor abnormalities) (Fig. 6). Indeed, the only observable differences were phospho-cofilin levels being lower in the male R6/2 mouse (cf. WT male; *P* < 0.05) and phospho-SSH1L being lower in male WT mice (vs female WT mice) (*P* < 0.05). At 10w (e.g. overt pathology), phospho-ROCK2 was higher (*P* < 0.05), while phospho-LIMK1 was lower (*P* < 0.01) in the female R6/2 mouse. PAK and cofilin were not affected by the R6/2 genotype, but levels of phospho-PAK were higher (*P* < 0.05) and phospho-cofilin was lower (*P* < 0.05) WT females *vs*. WT males.

Remarkably, the levels of phosphoSer138-profilin, which were substantially higher in the R6/2 mice than in the WT mice at the E14 stage (see Fig. 5), remained high in the R6/2 mouse at the 3w stage (*P* < 0.05), were comparable to levels in the WT mice at 5w of age, and by 10w were significantly lower in the R6/2 mice than in the WT mice (female: *P* < 0.01; male: *P* < 0.05) (Fig. 6f). This pattern was not influenced by the sex of the mouse.

## Discussion

Kinome analysis based on peptide arrays is a validated platform (Scholma et al. 2016) for identifying biochemical alterations in conditions as diverse as prion disease (Arsenault et al. 2012), Alzheimer’s disease (Hoozemans et al. 2012), cancer (Goel et al. 2018; Parikh and Peppelenbosch 2010; Labots et al. 2016), infectious diseases (Van Wyk et al. 2016; Robertson et al. 2014; Mulongo et al. 2014; Kindrachuk et al. 2014), and inflammation (Arsenault et al. 2013b, 2013a). Systematic quantitative proteomics—supported by mass spectrometry—based on striatal tissues from the R6/2 mouse (Hosp et al. 2017) and post-mortem HD patient samples (Ratovitski et al. 2016) revealed a widespread loss of protein function that implicates Rho proteins, actin cytoskeleton signaling, and mitochondria (Ratovitski et al. 2016), as well as proteins related to energy metabolism and cellular transport/cytoskeleton (Wegrzynowicz et al. 2012). A recent quantitative proteomic study implicated HTT as a critical regulator of neural injury response in adult mice suggesting its importance in neuronal survival and axon regeneration (Belin et al. 2015). Several high-throughput studies of HD using mouse models, post-mortem brain, and patient-derived stem cells also implicated dysregulation of actin signaling, including a loss of profilin expression at early stages of the disease process (DiProspero et al. 2004; Goldberg 2003; Heng et al. 2010; Lorincz and Zawistowski 2009; McQuade et al. 2014; Niwa et al. 2002; Burnett et al. 2008).

Our kinome analysis of striatal tissues identified several candidate pathways (see Supplementary Figs. 5–8) including the Akt/FOXO3 pathway that is neuroprotective in HD (Farina et al. 2017) and CDK2 (which we also observed in our NSC extracts, Fig. 1), thus suggesting a dysregulation of cell cycle regulatory proteins (Sang et al. 2014). We identified a loss of phosphorylation of mTOR and a corresponding increase in the phosphorylation of ULK1, which would indicate an activation of autophagy, likely in response to cellular stress (Rui et al. 2015), and our observed loss of RAF/MEK/CREB signalling over the symptomatic course in the R6/2 mice is consistent with a loss of ERK activation in cell death in models of HD (Bodai and Marsh 2012). ERK and Akt signalling deficits have been implicated in the loss of differentiation and neurite retraction in Q48- and Q89-expressing (but not Q16) PC12 cells (Song et al. 2002) and although these systems deserve to be characterized within the context of the R6/2 mouse, we focused on the Rho-Rac GTPase effector proteins, e.g. ROCK and PAK given our preliminary observations based on HC and HD NSC cultures. Although ROCK and PAK target unique substrates, there is abundant evidence that they both modulate LIMK-cofilin signaling and associated phenotypes. LIMK1 is highly expressed in the brain (Proschel et al. 1995) and the LIMK-cofilin association helps support the integrity and structure of dendritic spines (Linseman and Loucks 2008; Govek et al. 2005) as well as axonal growth (Heng et al. 2010; Koch et al. 2014). Part of cytoskeletal integrity might rely on the phosphorylation of cofilin-Ser3 by LIMK1, which inactivates cofilin and prevents its binding to actin (Yang et al. 1998). Loss of phosphorylation of cofilin-Ser3 (as we’ve seen in some of our samples) impairs cofilin function and monomeric actin-turnover in the cytoplasm leading to motility and morphological deficits, such as cell shrinkage (Munsie et al. 2012; Bravo-Cordero et al. 2013). The dephosphorylation of cofilin is not necessarily negative; indeed, during cell stress dephosphorylated cofilin can be sequestered as cofilin-actin rods, thus freeing up a pool of ATP bound to cofilin for critical cellular processes (Bernstein et al. 2006). Wildtype HTT helps localize these cofilin-actin rods to the nucleus, but these rods then disappear with the relief of the cellular stress; in contrast, mHTT induces a dominant, persistent nuclear cofilin-actin rod phenotype that triggers, amongst other events, an increase in calcium levels and cell death (Munsie et al. 2011).

Profilin exerts the opposite action to cofilin on actin and its polymerization, and can affect neuronal growth cone and synaptic plasticity (Birbach 2008). Yet the roles of these pathways are not as straightforward as anticipated. Indeed, the Rho and Rac pathways have been shown to exert mutual antagonism in N-Cadherin-mediated contact mechanisms in myoblasts, but sequential roles for these kinases are essential for contact communication in these same cells (Comunale et al. 2007). Interestingly, inhibition of either Rho or Rac elicit opposite effects of actin-based repair mechanisms in gastric epithelium (Aihara et al. 2018) and while Rho does not exert much influence on the leading edge of lamellipodia in rat adenocarcinoma cells, its inhibition does unmask a Rac-mediated facilitation of edge growth (El-Sibai et al. 2008). The phosphatase SSH1L also targets cofilin-Ser3, thus promoting cofilin (re)activation (Romarowski et al. 2015). As with Rho and Rac, the role of SSH1L in LIMK-cofilin-actin polymerization is viewed more as a context-dependent or sequence-dependent influence rather than simply as the phosphatase that targets cofilin.

A correlation between profilin inactivation and altered cytoskeletal dynamics could affect neurite morphology, given that actin microfilaments tend to be concentrated at the synaptic terminals, dendritic spines, and growth cones (Matus et al. 1982; Gordon-Weeks 1987). Although we observed changes in phosphorylation of both ROCK2 and PAK in the R62 mice at E14, we did not observe any corresponding change in the phosphorylation of their purported targets LIMK1 or cofilin (although we did observe a tendency for an increase in phosphoSer3-cofilin in E14 female R6/2 mice: *P* = 0.055; Fig. 5g). The phosphorylation of profilin was significantly higher at E14 in R6/2 mice compared to WT mice, regardless of sex, which suggests that the phosphorylation of ROCK2 (cf. the loss of phosphorylation of PAK) might be driving the phosphorylation of Ser138-profilin at this time point. This would presumably stabilize developmental dynamic actin structures and could help explain the overabundance of synaptic connectivity (likely due to a pruning defect) demonstrated elsewhere in a conditional knockout of Htt as well as a knock-in (Q175) mouse model of HD (McKinstry et al. 2014). Interestingly, these authors demonstrate that the loss of normal Htt function leads to an unanticipated early exaggerated increase in synapse formation; however, this phenotype is not sustainable and eventually leads to a loss of synaptic density by 5 weeks of age and a gliosis, but no neuronal death (McKinstry et al. 2014). Similarly, the expression of mHtt in the R6/1 mouse and in the Q7/Q111 knock-in mouse leads to a corticostriatal phenotype centered on deficits in cortical cell migration and electrophysiological properties, including a loss of long-term potentiation, a mechanism for strengthening synapses and critical for memory formation (Puigdellivol et al. 2015). This cortical phenotype precedes any loss of striatal synaptic integrity and associated motor deficits, and appears to be triggered by the loss (by two months of age) of Kalirin-7, a brain-specific Rho-guanine nucleotide exchange factor for Rac-like GTPases, that is expressed highly in dendritic spines of neuronal populations (Puigdellivol et al. 2015). The concurrent hyper- and hypo-phosphorylation of ROCK and PAK, respectively, observed at this time point (Fig. 5a, b) adds support to the notion of context-dependent and/or sequence-dependent influences of ROCK and PAK, as discussed in the previous paragraph.

Western blot analyses of components of the striatal ROCK2 and PAK pathways revealed dynamic changes with some level of similarity within sex/genotype across the postnatal time-points (Fig. 6). For example, the phosphorylation pattern of ROCK2 between the sexes and genotypes at 5w was more similar to the pattern observed at 3w than the pattern at 10w, while the pattern of phosphorylation of PAK and cofilin at 5w resembled more so that in the 10w sample set. Patterns of phospho-LIMK1 and phospho-SSH1L appeared to be more in flux at 5w. Recall, it is this age in the R6/2 mouse that striatal mHTT immunoreactivity is first detected (Davies et al. 1997) and it is this age in the Q175 mouse that is associated with a loss of synaptic density and increased gliosis (McKinstry et al. 2014). In our studies, this age also aligned with a change in the relative levels of phosphoSer138-profilin. Indeed, the levels of phosphoSer138-profilin were substantially higher in R6/2 mice at E14 and only slightly less so at 3w (again regardless of sex), but at 5w they were similar to levels in the WT mice and by 10w they were significantly lower than the levels in WT mice. While a screen of Rho pathway mRNA transcripts found a significant increase in *profilin* mRNA expression in autopsied HD patient cortical samples as well as in 13w-old pooled (male + female) R6/2 mouse striatum (but not at 4w), there was no corresponding change in profilin protein expression (Narayanan et al. 2016) and these authors did not explore the phosphorylation status of profilin. It was shown elsewhere that Y-27632, a rho-kinase (ROCK) inhibitor, blocks the phosphorylation of profilin, which binds actin as well as Htt (Shao et al. 2008), reduces intracellular aggregation of Htt (Pollitt et al. 2003), and inhibits Htt toxicity in *Drosophila* and motor deficits in mice (treatment began at age 4 weeks) (Pollitt et al. 2003; Li et al. 2009). We are unclear as to why profilin shifts from a phosphorylated to an unphosphorylated state between 5 and 10 weeks of age and how this might align with pathology. Perhaps the *hyper*phosphorylated state of profilin observed at the embryonic stage releases mHTT to localize with, amongst other proteins, perinuclear α-actinin-1-enriched stress fibers (Tousley et al. 2019) and trigger disruption of the nuclear lamina (Gasset-Rosa et al. 2017) (as suggested by our pathways analysis; see Supplementary Fig. 2) and transport via the nuclear pore complex (Grima et al. 2017). In keeping with a nuclear phenotype, susceptibility to DNA damage or the induction of genes for cell cycle re-entry and transition from G1 to S phase (downstream of mitochondrial stress and normally leading to apoptosis in neurons) has also been shown to be proportional to the CAG repeat lengths (Q30, Q45, Q65, and Q81) in isogenic embryonic stem cell lines (Ooi et al. 2019). Perhaps this *hyper*phosphorylated profilin destabilizes actin structures and interferes with DA signalling (Lin et al. 2001) as observed in the early stages of the disease progression (McCarthy et al. 2011; Zhang et al. 2010). Interestingly, our pathways analysis also reveals a ‘disinhibition of SNARE formation’ (Supplementary Fig. 2) as well as a loss of ‘transmission across chemical synapses’ (Supplementary Fig. 3). This could alter quantal DA release and trigger the hyperactivity observed in young R6/2 mice (which gradually disappears until the mice become hypoactive by 8w) (Carter et al. 1999). Whatever the mechanism, our data suggest an mHTT-induced developmental profilin phenotype. An additional a priori conclusion stemming from this study is that if the inconsistent phosphorylation profiles for LIMK1, cofilin, and/or SSH1L are contributing to the phosphorylation of Ser138-profilin and to the onset or development of the disease, then their roles are likely sex-dependent and either sequential or cascade-specific, as suggested elsewhere and discussed above. Yet, it is also possible that other regulators of the LIMK-cofilin pathways, such as the cofilin phosphatase PP2A (Pendleton et al. 2003) or the LIMK phosphatase PP1 (Vorster et al. 2011), could be exerting temporal or sequential influences. We also cannot discount the possibility of interference by the mHTT in the function of the WT HTT, e.g. transport and trafficking (Caviston et al. 2007; Gunawardena et al. 2003; Her and Goldstein 2008; Orr et al. 2008) as trafficking and recycling pathways were also identified in our analysis (Supplementary Fig. 3). Any of these are potential mechanistic contributors to the phosphoSer138-profilin phenotype and symptomatic progression, and certainly warrant being investigated further in terms of a sex-dependent influence on HD.

Our initial observation of a reduction in neurite length in HD NSCs is consistent with reports of reduced neurite length or abnormal dendritic branching in HD (Ferrante et al. 1991; Rong et al. 2006; Liu et al. 2014). Recently, human HD patient cells and mouse (Q140/Q140) striatum and primary neurons were shown to be less sensitive to growth factor stimulation and this reflected a disruption of a Rac1:p85(PI3K):α-actinin-2 complex (e.g. the mHTT does not interact with p85), which is enriched in striatal neurons and spines (Tousley et al. 2019). The loss of a stable complex could impact any growth factor-mediated, PI3K-dependent neurite outgrowth. A role for Rac1 and Rho GTPase signalling in the context of HD was also demonstrated in a yeast two-hybrid screen designed to identify HTT binding partners (Tourette et al. 2014). That study identified a number of candidates found in Rho GTPase family signaling, including Ezrin, several PI3K family members, and BAIAP2 (brain-specific angiogenesis inhibitor 1-associated protein 2). Functional assays demonstrated that mHTT interferes with BAIAP2-mediated filopodia-like protrusions in mouse embryonic fibroblasts (Tourette et al. 2014). The effect of mHTT on neurite outgrowth seems quite specific given that iPSCs derived from HD (Q77; Q109; Q180) and control (Q18; Q21; Q33) can all be differentiated toward a healthy cortical neuronal fate; but the CAG repeat length in HD iPSCs correlate directly with decreased neurite lengths, without any overt effect on branching morphology (Mehta et al. 2018). Neurite outgrowth is essential for the proper wiring of the nervous system during development and regeneration (Miller and Suter 2018), and cytoskeletal proteins—along with HTT (Burrus et al. 2020)—are critical in this process.

Finally, we address our observation that dysregulation of phosphoSer138-profilin in the R6/2 mouse model occurs long before the reported onset of phenotypic changes. Indeed, the phosphorylation of profilin at the embryonic stage suggests a developmental phenotype, potentially centered on a pruning defect, as discussed above. However, the progressive reduction in phosphoSer138-profilin at postnatal stages suggests a progressive defect in actin cytoarchitecture across the symptomatic course of the disease. This might involve exaggerated pruning, if one considers our observation of a loss of neurite length (e.g. enhanced retraction) observed in the HD NSCs as well as the massive pruning of glutamatergic terminals observed in the Q140 (Deng et al. 2013) and Q175 (Rothe et al. 2015) mouse models of HD, and the progressive loss of somatosensory cortical dendritic spine density over symptomatic stages of the R6/2 mouse (Murmu et al. 2013). This provides additional support for the suggestion that HD follows a developmental course centered on defects in cortical neurogenesis, axonal transport, and Golgi apparatus organization (Humbert 2010). Furthermore, our data support the suggestion that the HD genotype (e.g., 72 + CAG repeats) leads to defects through a loss-of-function mechanism as early as the neurulation stage (Haremaki et al. 2019).

The expression of WT HTT tends to increase with brain development (Marques Sousa and Humbert 2013; Bhide et al. 1996). Previous studies have shown that WT HTT associates with microtubules and is involved with transport in both anterograde and retrograde directions, whereas mHTT interferes with these processes, ultimately affecting brain development and/or causing neuronal dysfunction and death (Caviston et al. 2007; Gunawardena et al. 2003; Her and Goldstein 2008; Orr et al. 2008). The appearance of striatal mHTT [at 5 weeks: (Davies et al. 1997)] and the reduction in levels of functional WT HTT [by week 7: (Zhang et al. 2003)] in the R6/2 mouse likely exacerbates the defects in neuronal connectivity and transport, and expedites the course of symptomatology. Transport defects initially led to the suggestion that the R6/2 mouse and its rapidly progressing phenotype is likely a better reflection of juvenile onset HD (more likely when the CAG repeat expansion is in excess of 70) (Mangiarini et al. 1996). The critical role for HTT in the brain has also led to the suggestion that HD is a neurodevelopmental disorder, rather than simply an adult neurodegenerative disorder (Wiatr et al. 2018) and has also led to the assumption that mHTT carriers experience normal brain development, but that an emerging degenerative phase ultimately leads to the appearance of clinical symptoms. A recent study based on human organoids suggests that the CAG/glutamine repeat length in HTT regulates neurogenesis during early development (Zhang et al. 2019), while a repeat length below the disease threshold benefits brain structure and general intelligence among children aged 6–18 years of age (Lee et al. 2017). These same authors also reported that a higher repeat length (as long as it is below disease threshold) gives females an advantage on cortical thickness and intelligence.

## Conclusion

Overall, our observations suggest a potential sex-dependent influence on cofilin/LIMK1/SSH1L signaling in HD. We are re-assured that several of the observations made using the R6/2 mouse tissues corroborated our observations in the HD NSCs (even if only a single biological replicate). This is even more re-assuring given that these HD NSCs were from a Q45 donor, while the R6/2 mice are Q120 and many other works that we cite herein are based on equally or more aggressive Q77, Q109, Q140, Q175, Q180 etc. genotypes. We were concerned that our analyses did not identify any consistent changes in *metabolism*, be it energy or substrate, which are often identified in HD-related screens. Part of this could be viewed as a limitation of the interpretation of kinome platform. For example, in males at E14, we observed a significant downregulation [*P* = 0.002189] as well as a significant upregulation [*P* = 0.03161] of ‘metabolism of amino acids’. This apparent contradiction might simply reflect two distinct phosphopeptides being identified in the screen (one being upregulated, the other being downregulated) and the potentially different roles of the parent proteins in ‘metabolism of amino acids’. We also acknowledge that a limitation of our study is that the data remain correlational; however, they do suggest a biological mechanism implicating a progressive phosphoSer138-profilin phenotype. Perhaps more importantly, our observations suggest that the phosphoSer138-profilin phenotype emerges in the earliest stages of brain development, well before any manifestation of symptoms, providing for a clinically targetable and modifiable event. This warrants investigating whether this phenotype is causative and, if so, is it specific to the R6/2 mouse model of HD or is it generalizable across models (e.g., Q77, Q140, Q175) and/or in clinical HD. As importantly, both males and females need to be included in future studies so as to better define potential sex-dependent mechanisms in HD onset and progression.

## Electronic supplementary material

Below is the link to the electronic supplementary material.

Supplementary file1 Supplementary Figure 1: Schematic diagram showing the timeline of disease progression in R6/2 mouse brain [adapted from (Davies et al. 1997)]. The time is indicated in days for the embryonic (E) time points, ‘P0’ for ‘birth’, and weeks (w) for postnatal time points. The progression of phenotypes is indicated above the arrows. The large arrows indicate the time points selected for the current analysis. The small arrows indicate additional developmental time points significant in the disease progression. (TIF 1605 kb)

Supplementary file2 Supplementary Figure 2: Bar graph generated by InnateDB using the output from PIIKA2 (details in Materials and Methods). Hyper-phosphorylated (positive) values from both sexes for all time points were analyzed and highly represented pathways are shown here (names across the x-axis). The y-axis represents the log p-value. Note, Innate DB summarizes the output from many online platforms such as KEGG, Reactome, PID NCI, PID Biocarta, NetPath, and INOH. As such, it is possible that the same pathways might be a ‘hit’ on several platforms and, for example, "Meiosis" had equal high scores across several platforms, as did “Meiotic recombination”. For sake of clarity, we simply listed any such multiple hits a single time in the graph. (TIF 3184 kb)

Supplementary file3 Supplementary Figure 3: Bar graph generated by InnateDB using the output from PIIKA2 (details in Materials and Methods). Hypo-phosphorylated (negative) values from both sexes for all time points were analyzed and highly represented pathways are included here (names across x-axis). The y-axis represents the log p-value. (TIF 4156 kb)

Supplementary file4 Supplementary Figure 4: Molecular signaling involved in cytoskeletal organization: This illustration depicts the Rho-Rac signaling pathway, the key effectors, and the downstream targets that affect actin polymerization. The molecule labels represented in green and pink are kinases. External cues such as dopamine or transforming growth factor-β (TGF-β) can activate ROCK and PAK GTPases and trigger the phosphorylation and activation of LIMK, thus allowing the phosphorylation (and inactivation) of cofilin. Cofilin is dephosphorylated (reactivated) by the phosphatase SSH1L, which can be regulated by, for example, PI3K, ROS, and calcium (Ca2+). Profilin and cofilin are involved in maintaining the balance between filamentous (F) and globular (G) actin, both of which regulate actin polymerization. A star indicates a target in which a corresponding phosphopeptide was identified by kinome analysis. GPCR: G protein-coupled receptor; ROCK1/2: Rho-associated protein kinase 1/2; PAK: p21-activated kinase; LIMK1: Lim domain kinase 1; SSH1L: slingshot protein phosphatase 1; ROS: Reactive oxygen species; PI3K: Phosphoinositide 3´-kinase. (TIF 2845 kb)

Supplementary file9 Supplementary Figure 5: Changes in the Akt1/FoxO/CDK2 signaling pathway extracted from the kinome analysis of the R6/2 mice: (A) Fold-change heatmap across four time-points, e.g. E14, 3w, 5w, and 10w, in both sexes. The clustering was based on the time-points and the fold-change values. The color key represents positive values in red and negative values in green. The protein and the specific phosphosite are indicated at the top of each column. (B) Scatterplot of the fold-changes based on sex. Males are represented as squares and females as circles, with a different color assigned to each time-point, as indicated in the panel. (TIF 983 kb)

Supplementary file11 Supplementary Figure 6: Western blots of the proteins involved in CDK2 signaling in wild type (WT) and R6/2 mice. Note, that due to limited tissue samples across the ages, we focused our Western blotting on the 10 week (10w) time-point. The average densitometry and corresponding Western blots of phosphorylated to total (A, B) Akt1, (C, D) FoxO proteins, and (E, F) CDK2 are shown. The anti-phospho-FoxO antibody detects FoxO1, FoxO3a, and FoxO4. The different isoforms are identified by their relative mobility on SDS-PAGE (kDa: kiloDalton). Each value was initially normalized to expression of α-Tubulin in the corresponding lane. Sex was considered as a variable in the statistical analyses using two-way ANOVA and post hoc multiple comparisons. The data are presented as mean ± sem (n=3). *: P < 0.05. (TIF 963 kb)

Supplementary file12 Supplementary Figure 7: Changes in selected phosphorylated peptides in the Akt/mTOR/ULK1 cascade identified in the kinome analysis of the R6/2 mice: (A) Fold-change heatmap across four time-points, e.g. E14, 3w, 5w, and 10w, in both sexes. The clustering was based on the time-points and the fold-change values. The color key represents positive values in red and negative values in green. The names of the peptides and the phosphosite are indicated at the top of each column. (B) Scatterplot of the fold-changes based on sex. Males are represented as squares and females as circles, with a different color assigned to each time-point, as indicated in the panel. (TIF 872 kb)

Supplementary file13 Supplementary Figure 8: Changes in selected phosphorylated peptides in the RAF/MEK/CREB cascade identified in the kinome analysis of the R6/2 mice: (A) Fold-change heatmap across four time-points, e.g. E14, 3w, 5w, and 10w, in both sexes. The clustering was based on the time-points and the fold-change values. The color key represents positive values in red and negative values in green. The names of the peptides and the phosphosite are indicated at the top of each column. (B) Scatterplot of the fold-changes based on sex. Males are represented as squares and females as circles, with a different color assigned to each time-point, as indicated in the panel. (TIF 903 kb)

Supplementary file5 Supplementary Table 1 List of peptides spotted in array (XLSX 65 kb)

Supplementary file6 Supplementary Table 2 PIIKA-2 output with P and FC statistics (XLSX 462 kb)

Supplementary file7 Supplementary Table 3 InnateDB upregulated output (XLSX 1423 kb)

Supplementary file8 Supplementary Table 4 InnateDB downregulated output (XLSX 1232 kb)

## Data Availability

Available through public data repository.

## References

[CR1] Aihara E, Medina-Candelaria NM, Hanyu H, Matthis AL, Engevik KA, Gurniak CB, Witke W, Turner JR, Zhang T, Montrose MH (2018) Cell injury triggers actin polymerization to initiate epithelial restitution. J Cell Sci. 10.1242/jcs.216317 30072444 10.1242/jcs.216317 PMC6127731

[CR2] Angeli S, Shao J, Diamond MI (2010) F-actin binding regions on the androgen receptor and huntingtin increase aggregation and alter aggregate characteristics. PLoS ONE 5(2):e9053. 10.1371/journal.pone.0009053 20140226 10.1371/journal.pone.0009053 PMC2816219

[CR3] Arsenault RJ, Li Y, Maattanen P, Scruten E, Doig K, Potter A, Griebel P, Kusalik A, Napper S (2013) Altered Toll-like receptor 9 signaling in *Mycobacterium avium* subsp. paratuberculosis-infected bovine monocytes reveals potential therapeutic targets. Infect Immun 81(1):226–237. 10.1128/IAI.00785-12 23115040 10.1128/IAI.00785-12 PMC3536146

[CR4] Arsenault RJ, Li Y, Potter A, Griebel PJ, Kusalik A, Napper S (2012) Induction of ligand-specific PrP (C) signaling in human neuronal cells. Prion 6(5):477–488. 10.4161/pri.21914 22918447 10.4161/pri.21914 PMC3510852

[CR5] Arsenault RJ, Napper S, Kogut MH (2013) *Salmonella enterica* Typhimurium infection causes metabolic changes in chicken muscle involving AMPK, fatty acid and insulin/mTOR signaling. Vet Res 44:35. 10.1186/1297-9716-44-35 23682635 10.1186/1297-9716-44-35 PMC3663815

[CR6] Belin S, Nawabi H, Wang C, Tang S, Latremoliere A, Warren P, Schorle H, Uncu C, Woolf CJ, He Z, Steen JA (2015) Injury-induced decline of intrinsic regenerative ability revealed by quantitative proteomics. Neuron 86(4):1000–1014. 10.1016/j.neuron.2015.03.060 25937169 10.1016/j.neuron.2015.03.060 PMC4551425

[CR7] Berard A, Kroeker A, McQueen P, Coombs KM (2018) Methods and approaches to disease mechanisms using systems kinomics. Synth Syst Biotechnol 3(1):34–43. 10.1016/j.synbio.2017.12.004 29911197 10.1016/j.synbio.2017.12.004 PMC5884222

[CR8] Bernstein BW, Chen H, Boyle JA, Bamburg JR (2006) Formation of actin-ADF/cofilin rods transiently retards decline of mitochondrial potential and ATP in stressed neurons. Am J Physiol Cell Physiol 291(5):C828-839. 10.1152/ajpcell.00066.2006 16738008 10.1152/ajpcell.00066.2006

[CR9] Bhide PG, Day M, Sapp E, Schwarz C, Sheth A, Kim J, Young AB, Penney J, Golden J, Aronin N, DiFiglia M (1996) Expression of normal and mutant huntingtin in the developing brain. J Neurosci 16(17):5523–5535 8757264 10.1523/JNEUROSCI.16-17-05523.1996 PMC6578889

[CR10] Birbach A (2008) Profilin, a multi-modal regulator of neuronal plasticity. BioEssays 30(10):994–1002. 10.1002/bies.20822 18798527 10.1002/bies.20822

[CR11] Bodai L, Marsh JL (2012) A novel target for Huntington’s disease: ERK at the crossroads of signaling. The ERK signaling pathway is implicated in Huntington’s disease and its upregulation ameliorates pathology. BioEssays 34(2):142–148. 10.1002/bies.201100116 22334892 10.1002/bies.201100116 PMC3711381

[CR12] Bode FJ, Stephan M, Suhling H, Pabst R, Straub RH, Raber KA, Bonin M, Nguyen HP, Riess O, Bauer A, Sjoberg C, Petersen A, von Horsten S (2008) Sex differences in a transgenic rat model of Huntington’s disease: decreased 17beta-estradiol levels correlate with reduced numbers of DARPP32+ neurons in males. Hum Mol Genet 17(17):2595–2609. 10.1093/hmg/ddn159 18502785 10.1093/hmg/ddn159

[CR13] Bravo-Cordero JJ, Magalhaes MA, Eddy RJ, Hodgson L, Condeelis J (2013) Functions of cofilin in cell locomotion and invasion. Nat Rev Mol Cell Biol 14(7):405–415. 10.1038/nrm3609 23778968 10.1038/nrm3609 PMC3878614

[CR14] Breuer K, Foroushani AK, Laird MR, Chen C, Sribnaia A, Lo R, Winsor GL, Hancock RE, Brinkman FS, Lynn DJ (2013) InnateDB: systems biology of innate immunity and beyond–recent updates and continuing curation. Nucleic Acids Res 41(Database issue):D1228–D1233. 10.1093/nar/gks1147 23180781 10.1093/nar/gks1147 PMC3531080

[CR15] Burnett BG, Andrews J, Ranganathan S, Fischbeck KH, Di Prospero NA (2008) Expression of expanded polyglutamine targets profilin for degradation and alters actin dynamics. Neurobiol Dis 30(3):365–374. 10.1016/j.nbd.2008.02.007 18417352 10.1016/j.nbd.2008.02.007 PMC2442575

[CR16] Burrus CJ, McKinstry SU, Kim N, Ozlu MI, Santoki AV, Fang FY, Ma A, Karadeniz YB, Worthington AK, Dragatsis I, Zeitlin S, Yin HH, Eroglu C (2020) Striatal Projection Neurons Require Huntingtin for Synaptic Connectivity and Survival. Cell Rep 30(3):642e646-657e646. 10.1016/j.celrep.2019.12.069 31968243 10.1016/j.celrep.2019.12.069 PMC7025500

[CR17] Carter RJ, Lione LA, Humby T, Mangiarini L, Mahal A, Bates GP, Dunnett SB, Morton AJ (1999) Characterization of progressive motor deficits in mice transgenic for the human Huntington’s disease mutation. J Neurosci 19(8):3248–3257 10191337 10.1523/JNEUROSCI.19-08-03248.1999 PMC6782264

[CR18] Caviston JP, Ross JL, Antony SM, Tokito M, Holzbaur EL (2007) Huntingtin facilitates dynein/dynactin-mediated vesicle transport. Proc Natl Acad Sci USA 104(24):10045–10050. 10.1073/pnas.0610628104 17548833 10.1073/pnas.0610628104 PMC1891230

[CR19] Cepeda C, Murphy KP, Parent M, Levine MS (2014) The role of dopamine in Huntington’s disease. Prog Brain Res 211:235–254. 10.1016/B978-0-444-63425-2.00010-6 24968783 10.1016/B978-0-444-63425-2.00010-6 PMC4409123

[CR20] Chen X, Grisham W, Arnold AP (2009) X chromosome number causes sex differences in gene expression in adult mouse striatum. Eur J Neurosci 29(4):768–776. 10.1111/j.1460-9568.2009.06610.x 19250439 10.1111/j.1460-9568.2009.06610.x

[CR21] Comunale F, Causeret M, Favard C, Cau J, Taulet N, Charrasse S, Gauthier-Rouviere C (2007) Rac1 and RhoA GTPases have antagonistic functions during N-cadherin-dependent cell-cell contact formation in C2C12 myoblasts. Biol Cell 99(9):503–517. 10.1042/BC20070011 17459003 10.1042/BC20070011

[CR22] Culver BP, Savas JN, Park SK, Choi JH, Zheng S, Zeitlin SO, Yates JR 3rd, Tanese N (2012) Proteomic analysis of wild-type and mutant huntingtin-associated proteins in mouse brains identifies unique interactions and involvement in protein synthesis. J Biol Chem 287(26):21599–21614. 10.1074/jbc.M112.359307 22556411 10.1074/jbc.M112.359307 PMC3381125

[CR23] Davies SW, Turmaine M, Cozens BA, DiFiglia M, Sharp AH, Ross CA, Scherzinger E, Wanker EE, Mangiarini L, Bates GP (1997) Formation of neuronal intranuclear inclusions underlies the neurological dysfunction in mice transgenic for the HD mutation. Cell 90(3):537–548. 10.1016/s0092-8674(00)80513-9 9267033 10.1016/s0092-8674(00)80513-9

[CR24] Deng YP, Wong T, Bricker-Anthony C, Deng B, Reiner A (2013) Loss of corticostriatal and thalamostriatal synaptic terminals precedes striatal projection neuron pathology in heterozygous Q140 Huntington’s disease mice. Neurobiol Dis 60:89–107. 10.1016/j.nbd.2013.08.009 23969239 10.1016/j.nbd.2013.08.009 PMC3808190

[CR25] DiProspero NA, Chen EY, Charles V, Plomann M, Kordower JH, Tagle DA (2004) Early changes in Huntington’s disease patient brains involve alterations in cytoskeletal and synaptic elements. J Neurocytol 33(5):517–533. 10.1007/s11068-004-0514-8 15906159 10.1007/s11068-004-0514-8

[CR26] El-Sibai M, Pertz O, Pang H, Yip SC, Lorenz M, Symons M, Condeelis JS, Hahn KM, Backer JM (2008) RhoA/ROCK-mediated switching between Cdc42- and Rac1-dependent protrusion in MTLn3 carcinoma cells. Exp Cell Res 314(7):1540–1552. 10.1016/j.yexcr.2008.01.016 18316075 10.1016/j.yexcr.2008.01.016 PMC2677995

[CR27] Farina F, Lambert E, Commeau L, Lejeune FX, Roudier N, Fonte C, Parker JA, Boddaert J, Verny M, Baulieu EE, Neri C (2017) The stress response factor daf-16/FOXO is required for multiple compound families to prolong the function of neurons with Huntington’s disease. Sci Rep 7(1):4014. 10.1038/s41598-017-04256-w 28638078 10.1038/s41598-017-04256-w PMC5479833

[CR28] Ferrante RJ, Kowall NW, Richardson EP Jr (1991) Proliferative and degenerative changes in striatal spiny neurons in Huntington’s disease: a combined study using the section-Golgi method and calbindin D28k immunocytochemistry. J Neurosci 11(12):3877–3887 1836019 10.1523/JNEUROSCI.11-12-03877.1991 PMC6575286

[CR29] Gasset-Rosa F, Chillon-Marinas C, Goginashvili A, Atwal RS, Artates JW, Tabet R, Wheeler VC, Bang AG, Cleveland DW, Lagier-Tourenne C (2017) Polyglutamine-expanded huntingtin exacerbates age-related disruption of nuclear integrity and nucleocytoplasmic transport. Neuron 94(1):48e44-57e44. 10.1016/j.neuron.2017.03.027 28384474 10.1016/j.neuron.2017.03.027 PMC5479704

[CR30] Goel RK, Paczkowska M, Reimand J, Napper S, Lukong KE (2018) Phosphoproteomics analysis identifies novel candidate substrates of the non-receptor tyrosine kinase, SRMS. Mol Cell Proteomics. 10.1074/mcp.RA118.000643 29496907 10.1074/mcp.RA118.000643 PMC5930402

[CR31] Goldberg JL (2003) How does an axon grow? Genes Dev 17(8):941–958. 10.1101/gad.1062303 12704078 10.1101/gad.1062303

[CR32] Gordon-Weeks PR (1987) The cytoskeletons of isolated, neuronal growth cones. Neuroscience 21(3):977–989 2888041 10.1016/0306-4522(87)90052-2

[CR33] Govek EE, Newey SE, Van Aelst L (2005) The role of the Rho GTPases in neuronal development. Genes Dev 19(1):1–49. 10.1101/gad.1256405 15630019 10.1101/gad.1256405

[CR34] Grima JC, Daigle JG, Arbez N, Cunningham KC, Zhang K, Ochaba J, Geater C, Morozko E, Stocksdale J, Glatzer JC, Pham JT, Ahmed I, Peng Q, Wadhwa H, Pletnikova O, Troncoso JC, Duan W, Snyder SH, Ranum LPW, Thompson LM, Lloyd TE, Ross CA, Rothstein JD (2017) Mutant huntingtin disrupts the nuclear pore complex. Neuron 94(1):93e106-107e106. 10.1016/j.neuron.2017.03.023 28384479 10.1016/j.neuron.2017.03.023 PMC5595097

[CR35] Gunawardena S, Her LS, Brusch RG, Laymon RA, Niesman IR, Gordesky-Gold B, Sintasath L, Bonini NM, Goldstein LS (2003) Disruption of axonal transport by loss of huntingtin or expression of pathogenic polyQ proteins in *Drosophila*. Neuron 40(1):25–40. 10.1016/s0896-6273(03)00594-4 14527431 10.1016/s0896-6273(03)00594-4

[CR36] Haremaki T, Metzger JJ, Rito T, Ozair MZ, Etoc F, Brivanlou AH (2019) Self-organizing neuruloids model developmental aspects of Huntington’s disease in the ectodermal compartment. Nat Biotechnol 37(10):1198–1208. 10.1038/s41587-019-0237-5 31501559 10.1038/s41587-019-0237-5

[CR37] Heng JI, Chariot A, Nguyen L (2010) Molecular layers underlying cytoskeletal remodelling during cortical development. Trends Neurosci 33(1):38–47. 10.1016/j.tins.2009.09.003 19837469 10.1016/j.tins.2009.09.003

[CR38] Her LS, Goldstein LS (2008) Enhanced sensitivity of striatal neurons to axonal transport defects induced by mutant huntingtin. J Neurosci 28(50):13662–13672. 10.1523/JNEUROSCI.4144-08.2008 19074039 10.1523/JNEUROSCI.4144-08.2008 PMC6671757

[CR39] Hoozemans JJ, Hilhorst R, Ruijtenbeek R, Rozemuller AJ, van der Vies SM (2012) Protein kinase activity profiling of postmortem human brain tissue. Neuro-degener Dis 10(1–4):46–48. 10.1159/000335914 10.1159/000335914 22343098

[CR40] Hornbeck PV, Kornhauser JM, Tkachev S, Zhang B, Skrzypek E, Murray B, Latham V, Sullivan M (2012) PhosphoSitePlus: a comprehensive resource for investigating the structure and function of experimentally determined post-translational modifications in man and mouse. Nucleic Acids Res 40(Database issue):D261–D270. 10.1093/nar/gkr1122 22135298 10.1093/nar/gkr1122 PMC3245126

[CR41] Hosp F, Gutierrez-Angel S, Schaefer MH, Cox J, Meissner F, Hipp MS, Hartl FU, Klein R, Dudanova I, Mann M (2017) Spatiotemporal proteomic profiling of Huntington’s disease inclusions reveals widespread loss of protein function. Cell Rep 21(8):2291–2303. 10.1016/j.celrep.2017.10.097 29166617 10.1016/j.celrep.2017.10.097 PMC5714591

[CR42] Humbert S (2010) Is Huntington disease a developmental disorder? EMBO Rep 11(12):899. 10.1038/embor.2010.182 21102640 10.1038/embor.2010.182 PMC2999873

[CR43] Jalal S, Arsenault R, Potter AA, Babiuk LA, Griebel PJ, Napper S (2009) Genome to kinome: species-specific peptide arrays for kinome analysis. Sci Signal. 10.1126/scisignal.254pl1 19155530 10.1126/scisignal.254pl1

[CR44] Julian L, Olson MF (2014) Rho-associated coiled-coil containing kinases (ROCK): structure, regulation, and functions. Small GTPases 5:e29846. 10.4161/sgtp.29846 25010901 10.4161/sgtp.29846 PMC4114931

[CR45] Kindrachuk J, Wahl-Jensen V, Safronetz D, Trost B, Hoenen T, Arsenault R, Feldmann F, Traynor D, Postnikova E, Kusalik A, Napper S, Blaney JE, Feldmann H, Jahrling PB (2014) Ebola virus modulates transforming growth factor beta signaling and cellular markers of mesenchyme-like transition in hepatocytes. J Virol 88(17):9877–9892. 10.1128/JVI.01410-14 24942569 10.1128/JVI.01410-14 PMC4136307

[CR46] Koch JC, Tonges L, Barski E, Michel U, Bahr M, Lingor P (2014) ROCK2 is a major regulator of axonal degeneration, neuronal death and axonal regeneration in the CNS. Cell Death Dis 5:e1225. 10.1038/cddis.2014.191 24832597 10.1038/cddis.2014.191 PMC4047920

[CR47] Koller WC, Barr A, Biary N (1982) Estrogen treatment of dyskinetic disorders. Neurology 32(5):547–549. 10.1212/wnl.32.5.547 7200210 10.1212/wnl.32.5.547

[CR48] Labots M, Gotink KJ, Dekker H, Azijli K, van der Mijn JC, Huijts CM, Piersma SR, Jimenez CR, Verheul HM (2016) Evaluation of a tyrosine kinase peptide microarray for tyrosine kinase inhibitor therapy selection in cancer. Exp Mol Med 48(12):e279. 10.1038/emm.2016.114 27980342 10.1038/emm.2016.114 PMC5192072

[CR49] Lee JK, Ding Y, Conrad AL, Cattaneo E, Epping E, Mathews K, Gonzalez-Alegre P, Cahill L, Magnotta V, Schlaggar BL, Perlmutter JS, Kim RE, Dawson JD, Nopoulos P (2017) Sex-specific effects of the Huntington gene on normal neurodevelopment. J Neurosci Res 95(1–2):398–408. 10.1002/jnr.23980 27870408 10.1002/jnr.23980 PMC5729280

[CR50] Li M, Huang Y, Ma AA, Lin E, Diamond MI (2009) Y-27632 improves rotarod performance and reduces huntingtin levels in R6/2 mice. Neurobiol Dis 36(3):413–420. 10.1016/j.nbd.2009.06.011 19591939 10.1016/j.nbd.2009.06.011

[CR51] Lin R, Karpa K, Kabbani N, Goldman-Rakic P, Levenson R (2001) Dopamine D2 and D3 receptors are linked to the actin cytoskeleton via interaction with filamin A. Proc Natl Acad Sci USA 98(9):5258–5263. 10.1073/pnas.011538198 11320256 10.1073/pnas.011538198 PMC33197

[CR52] Linseman DA, Loucks FA (2008) Diverse roles of Rho family GTPases in neuronal development, survival, and death. Front Biosci 13:657–676. 10.2741/2710 17981578 10.2741/2710

[CR53] Liu Y, Xue Y, Ridley S, Zhang D, Rezvani K, Fu XD, Wang H (2014) Direct reprogramming of Huntington’s disease patient fibroblasts into neuron-like cells leads to abnormal neurite outgrowth, increased cell death, and aggregate formation. PLoS ONE 9(10):e109621. 10.1371/journal.pone.0109621 25275533 10.1371/journal.pone.0109621 PMC4183653

[CR54] Lorincz MT, Zawistowski VA (2009) Expanded CAG repeats in the murine Huntington’s disease gene increases neuronal differentiation of embryonic and neural stem cells. Mol Cell Neurosci 40(1):1–13. 10.1016/j.mcn.2008.06.004 18625318 10.1016/j.mcn.2008.06.004 PMC2666278

[CR55] Maattanen P, Taschuk R, Ross L, Marciniuk K, Bertram L, Potter A, Cashman NR, Napper S (2013) PrP(Sc)-specific antibodies do not induce prion disease or misfolding of PrP(C) in highly susceptible Tga20 mice. Prion 7(5):434–439. 10.4161/pri.26639 24105298 10.4161/pri.26639 PMC4134347

[CR56] Mangiarini L, Sathasivam K, Seller M, Cozens B, Harper A, Hetherington C, Lawton M, Trottier Y, Lehrach H, Davies SW, Bates GP (1996) Exon 1 of the HD gene with an expanded CAG repeat is sufficient to cause a progressive neurological phenotype in transgenic mice. Cell 87(3):493–506 8898202 10.1016/s0092-8674(00)81369-0

[CR57] Marques Sousa C, Humbert S (2013) Huntingtin: here, there, everywhere! J Huntingtons Dis 2(4):395–403. 10.3233/JHD-130082 25062728 10.3233/JHD-130082

[CR58] Matus A, Ackermann M, Pehling G, Byers HR, Fujiwara K (1982) High actin concentrations in brain dendritic spines and postsynaptic densities. Proc Natl Acad Sci USA 79(23):7590–7594 6760199 10.1073/pnas.79.23.7590 PMC347386

[CR59] McCarthy DM, Zhang X, Darnell SB, Sangrey GR, Yanagawa Y, Sadri-Vakili G, Bhide PG (2011) Cocaine alters BDNF expression and neuronal migration in the embryonic mouse forebrain. J Neurosci 31(38):13400–13411. 10.1523/JNEUROSCI.2944-11.2011 21940433 10.1523/JNEUROSCI.2944-11.2011 PMC3182852

[CR60] McKinstry SU, Karadeniz YB, Worthington AK, Hayrapetyan VY, Ozlu MI, Serafin-Molina K, Risher WC, Ustunkaya T, Dragatsis I, Zeitlin S, Yin HH, Eroglu C (2014) Huntingtin is required for normal excitatory synapse development in cortical and striatal circuits. J Neurosci 34(28):9455–9472. 10.1523/JNEUROSCI.4699-13.2014 25009276 10.1523/JNEUROSCI.4699-13.2014 PMC4087216

[CR61] McQuade LR, Balachandran A, Scott HA, Khaira S, Baker MS, Schmidt U (2014) Proteomics of Huntington’s disease-affected human embryonic stem cells reveals an evolving pathology involving mitochondrial dysfunction and metabolic disturbances. J Proteome Res 13(12):5648–5659. 10.1021/pr500649m 25316320 10.1021/pr500649m

[CR62] Mehta SR, Tom CM, Wang Y, Bresee C, Rushton D, Mathkar PP, Tang J, Mattis VB (2018) Human Huntington’s disease iPSC-derived cortical neurons display altered transcriptomics, morphology, and maturation. Cell Rep 25(4):1081e1086-1096e1086. 10.1016/j.celrep.2018.09.076 30355486 10.1016/j.celrep.2018.09.076

[CR63] Miller KE, Suter DM (2018) An integrated cytoskeletal model of neurite outgrowth. Front Cell Neurosci 12:447. 10.3389/fncel.2018.00447 30534055 10.3389/fncel.2018.00447 PMC6275320

[CR64] Mulongo M, Prysliak T, Scruten E, Napper S, Perez-Casal J (2014) *In vitro* infection of bovine monocytes with *Mycoplasma bovis* delays apoptosis and suppresses production of gamma interferon and tumor necrosis factor alpha but not interleukin-10. Infect Immun 82(1):62–71. 10.1128/IAI.00961-13 24126524 10.1128/IAI.00961-13 PMC3911867

[CR65] Munsie L, Caron N, Atwal RS, Marsden I, Wild EJ, Bamburg JR, Tabrizi SJ, Truant R (2011) Mutant huntingtin causes defective actin remodeling during stress: defining a new role for transglutaminase 2 in neurodegenerative disease. Hum Mol Genet 20(10):1937–1951. 10.1093/hmg/ddr075 21355047 10.1093/hmg/ddr075 PMC3080606

[CR66] Munsie LN, Desmond CR, Truant R (2012) Cofilin nuclear-cytoplasmic shuttling affects cofilin-actin rod formation during stress. J Cell Sci 125(Pt 17):3977–3988. 10.1242/jcs.097667 22623727 10.1242/jcs.097667

[CR67] Murmu RP, Li W, Holtmaat A, Li JY (2013) Dendritic spine instability leads to progressive neocortical spine loss in a mouse model of Huntington’s disease. J Neurosci 33(32):12997–13009. 10.1523/JNEUROSCI.5284-12.2013 23926255 10.1523/JNEUROSCI.5284-12.2013 PMC6619731

[CR68] Narayanan KL, Chopra V, Rosas HD, Malarick K, Hersch S (2016) Rho kinase pathway alterations in the brain and leukocytes in Huntington’s disease. Mol Neurobiol 53(4):2132–2140. 10.1007/s12035-015-9147-9 25941073 10.1007/s12035-015-9147-9 PMC4823347

[CR69] Niwa R, Nagata-Ohashi K, Takeichi M, Mizuno K, Uemura T (2002) Control of actin reorganization by Slingshot, a family of phosphatases that dephosphorylate ADF/cofilin. Cell 108(2):233–246 11832213 10.1016/s0092-8674(01)00638-9

[CR70] Ooi J, Langley SR, Xu X, Utami KH, Sim B, Huang Y, Harmston NP, Tay YL, Ziaei A, Zeng R, Low D, Aminkeng F, Sobota RM, Ginhoux F, Petretto E, Pouladi MA (2019) Unbiased profiling of isogenic huntington disease hPSC-derived CNS and peripheral cells reveals strong cell-type specificity of CAG length effects. Cell Rep 26(9):2494e2497-2508e2497. 10.1016/j.celrep.2019.02.008 30811996 10.1016/j.celrep.2019.02.008

[CR71] Orr AL, Li S, Wang CE, Li H, Wang J, Rong J, Xu X, Mastroberardino PG, Greenamyre JT, Li XJ (2008) N-terminal mutant huntingtin associates with mitochondria and impairs mitochondrial trafficking. J Neurosci 28(11):2783–2792. 10.1523/JNEUROSCI.0106-08.2008 18337408 10.1523/JNEUROSCI.0106-08.2008 PMC2652473

[CR72] Parikh K, Peppelenbosch MP (2010) Kinome profiling of clinical cancer specimens. Cancer Res 70(7):2575–2578. 10.1158/0008-5472.CAN-09-3989 20332226 10.1158/0008-5472.CAN-09-3989

[CR73] Pendleton A, Pope B, Weeds A, Koffer A (2003) Latrunculin B or ATP depletion induces cofilin-dependent translocation of actin into nuclei of mast cells. J Biol Chem 278(16):14394–14400. 10.1074/jbc.M206393200 12566455 10.1074/jbc.M206393200

[CR74] Pierzynowska K, Gaffke L, Cyske Z, Wegrzyn G (2019) Genistein induces degradation of mutant huntingtin in fibroblasts from Huntington’s disease patients. Metab Brain Dis 34(3):715–720. 10.1007/s11011-019-00405-4 30850940 10.1007/s11011-019-00405-4 PMC6520327

[CR75] Pollitt SK, Pallos J, Shao J, Desai UA, Ma AA, Thompson LM, Marsh JL, Diamond MI (2003) A rapid cellular FRET assay of polyglutamine aggregation identifies a novel inhibitor. Neuron 40(4):685–694. 10.1016/s0896-6273(03)00697-4 14622574 10.1016/s0896-6273(03)00697-4

[CR76] Poplawski GHD, Kawaguchi R, Van Niekerk E, Lu P, Mehta N, Canete P, Lie R, Dragatsis I, Meves JM, Zheng B, Coppola G, Tuszynski MH (2020) Injured adult neurons regress to an embryonic transcriptional growth state. Nature. 10.1038/s41586-020-2200-5 32376949 10.1038/s41586-020-2200-5

[CR77] Proschel C, Blouin MJ, Gutowski NJ, Ludwig R, Noble M (1995) Limk1 is predominantly expressed in neural tissues and phosphorylates serine, threonine and tyrosine residues in vitro. Oncogene 11(7):1271–1281 7478547

[CR78] Puigdellivol M, Cherubini M, Brito V, Giralt A, Suelves N, Ballesteros J, Zamora-Moratalla A, Martin ED, Eipper BA, Alberch J, Gines S (2015) A role for Kalirin-7 in corticostriatal synaptic dysfunction in Huntington’s disease. Hum Mol Genet 24(25):7265–7285. 10.1093/hmg/ddv426 26464483 10.1093/hmg/ddv426 PMC4664166

[CR79] Ratovitski T, Chaerkady R, Kammers K, Stewart JC, Zavala A, Pletnikova O, Troncoso JC, Rudnicki DD, Margolis RL, Cole RN, Ross CA (2016) Quantitative proteomic analysis reveals similarities between Huntington’s disease (HD) and Huntington’s disease-like 2 (HDL2) human brains. J Proteome Res 15(9):3266–3283. 10.1021/acs.jproteome.6b00448 27486686 10.1021/acs.jproteome.6b00448 PMC5555151

[CR80] Robertson AJ, Trost B, Scruten E, Robertson T, Mostajeran M, Connor W, Kusalik A, Griebel P, Napper S (2014) Identification of developmentally-specific kinotypes and mechanisms of Varroa mite resistance through whole-organism, kinome analysis of honeybee. Front Genet 5:139. 10.3389/fgene.2014.00139 24904639 10.3389/fgene.2014.00139 PMC4033134

[CR81] Romarowski A, Battistone MA, La Spina FA, Puga Molina Ldel C, Luque GM, Vitale AM, Cuasnicu PS, Visconti PE, Krapf D, Buffone MG (2015) PKA-dependent phosphorylation of LIMK1 and Cofilin is essential for mouse sperm acrosomal exocytosis. Dev Biol 405(2):237–249. 10.1016/j.ydbio.2015.07.008 26169470 10.1016/j.ydbio.2015.07.008 PMC4546557

[CR82] Rong J, McGuire JR, Fang ZH, Sheng G, Shin JY, Li SH, Li XJ (2006) Regulation of intracellular trafficking of huntingtin-associated protein-1 is critical for TrkA protein levels and neurite outgrowth. J Neurosci 26(22):6019–6030. 10.1523/JNEUROSCI.1251-06.2006 16738245 10.1523/JNEUROSCI.1251-06.2006 PMC6675209

[CR83] Roos RA, Vegter-van der Vlis M, Hermans J, Elshove HM, Moll AC, van de Kamp JJ, Bruyn GW (1991) Age at onset in Huntington’s disease: effect of line of inheritance and patient’s sex. J Med Genet 28(8):515–519 1833547 10.1136/jmg.28.8.515 PMC1016978

[CR84] Ross CA, Tabrizi SJ (2011) Huntington’s disease: from molecular pathogenesis to clinical treatment. Lancet Neurol 10(1):83–98. 10.1016/S1474-4422(10)70245-3 21163446 10.1016/S1474-4422(10)70245-3

[CR85] Rothe T, Deliano M, Wojtowicz AM, Dvorzhak A, Harnack D, Paul S, Vagner T, Melnick I, Stark H, Grantyn R (2015) Pathological gamma oscillations, impaired dopamine release, synapse loss and reduced dynamic range of unitary glutamatergic synaptic transmission in the striatum of hypokinetic Q175 Huntington mice. Neuroscience 311:519–538. 10.1016/j.neuroscience.2015.10.039 26546830 10.1016/j.neuroscience.2015.10.039

[CR86] Roze E, Cahill E, Martin E, Bonnet C, Vanhoutte P, Betuing S, Caboche J (2011) Huntington’s disease and striatal signaling. Front Neuroanat 5:55. 10.3389/fnana.2011.00055 22007160 10.3389/fnana.2011.00055 PMC3188786

[CR87] Rui YN, Xu Z, Patel B, Cuervo AM, Zhang S (2015) HTT/Huntingtin in selective autophagy and Huntington disease: A foe or a friend within? Autophagy 11(5):858–860. 10.1080/15548627.2015.1039219 25985010 10.1080/15548627.2015.1039219 PMC4509439

[CR88] Sang T, Cao Q, Wang Y, Liu F, Chen S (2014) Overexpression or silencing of FOXO3a affects proliferation of endothelial progenitor cells and expression of cell cycle regulatory proteins. PLoS ONE 9(8):e101703. 10.1371/journal.pone.0101703 25093499 10.1371/journal.pone.0101703 PMC4122338

[CR89] Saudou F, Humbert S (2016) The Biology of Huntingtin. Neuron 89(5):910–926. 10.1016/j.neuron.2016.02.003 26938440 10.1016/j.neuron.2016.02.003

[CR90] Scholma J, Fuhler GM, Joore J, Hulsman M, Schivo S, List AF, Reinders MJ, Peppelenbosch MP, Post JN (2016) Improved intra-array and interarray normalization of peptide microarray phosphorylation for phosphorylome and kinome profiling by rational selection of relevant spots. Sci Rep 6:26695. 10.1038/srep26695 27225531 10.1038/srep26695 PMC4881024

[CR91] Shao J, Welch WJ, Diprospero NA, Diamond MI (2008) Phosphorylation of profilin by ROCK1 regulates polyglutamine aggregation. Mol Cell Biol 28(17):5196–5208. 10.1128/MCB.00079-08 18573880 10.1128/MCB.00079-08 PMC2519718

[CR92] Song C, Perides G, Liu YF (2002) Expression of full-length polyglutamine-expanded Huntingtin disrupts growth factor receptor signaling in rat pheochromocytoma (PC12) cells. J Biol Chem 277(8):6703–6707. 10.1074/jbc.M110338200 11733534 10.1074/jbc.M110338200

[CR93] Tourette C, Li B, Bell R, O’Hare S, Kaltenbach LS, Mooney SD, Hughes RE (2014) A large scale Huntingtin protein interaction network implicates Rho GTPase signaling pathways in Huntington disease. J Biol Chem 289(10):6709–6726. 10.1074/jbc.M113.523696 24407293 10.1074/jbc.M113.523696 PMC3945331

[CR94] Tousley A, Iuliano M, Weisman E, Sapp E, Richardson H, Vodicka P, Alexander J, Aronin N, DiFiglia M, Kegel-Gleason KB (2019) Huntingtin associates with the actin cytoskeleton and alpha-actinin isoforms to influence stimulus dependent morphology changes. PLoS ONE 14(2):e0212337. 10.1371/journal.pone.0212337 30768638 10.1371/journal.pone.0212337 PMC6377189

[CR95] Trost B, Arsenault R, Griebel P, Napper S, Kusalik A (2013) DAPPLE: a pipeline for the homology-based prediction of phosphorylation sites. Bioinformatics 29(13):1693–1695. 10.1093/bioinformatics/btt265 23658419 10.1093/bioinformatics/btt265

[CR96] Trost B, Kindrachuk J, Maattanen P, Napper S, Kusalik A (2013) PIIKA 2: an expanded, web-based platform for analysis of kinome microarray data. PLoS ONE 8(11):e80837. 10.1371/journal.pone.0080837 24312246 10.1371/journal.pone.0080837 PMC3843739

[CR97] Trushina E, Dyer RB, Badger JD 2nd, Ure D, Eide L, Tran DD, Vrieze BT, Legendre-Guillemin V, McPherson PS, Mandavilli BS, Van Houten B, Zeitlin S, McNiven M, Aebersold R, Hayden M, Parisi JE, Seeberg E, Dragatsis I, Doyle K, Bender A, Chacko C, McMurray CT (2004) Mutant huntingtin impairs axonal trafficking in mammalian neurons in vivo and in vitro. Mol Cell Biol 24(18):8195–8209. 10.1128/MCB.24.18.8195-8209.2004 15340079 10.1128/MCB.24.18.8195-8209.2004 PMC515048

[CR98] Van Wyk B, Snider M, Scruten E, van Drunen Littel-van den Hurk S, Napper S (2016) Induction of functional interferon alpha and gamma responses during acute infection of cattle with non-cytopathic bovine viral diarrhea virus. Vet Microbiol 195:104–114. 10.1016/j.vetmic.2016.09.015 27771055 10.1016/j.vetmic.2016.09.015

[CR99] Voorn P, Kalsbeek A, Jorritsma-Byham B, Groenewegen HJ (1988) The pre- and postnatal development of the dopaminergic cell groups in the ventral mesencephalon and the dopaminergic innervation of the striatum of the rat. Neuroscience 25(3):857–887. 10.1016/0306-4522(88)90041-3 3405431 10.1016/0306-4522(88)90041-3

[CR100] Vorster PJ, Guo J, Yoder A, Wang W, Zheng Y, Xu X, Yu D, Spear M, Wu Y (2011) LIM kinase 1 modulates cortical actin and CXCR4 cycling and is activated by HIV-1 to initiate viral infection. J Biol Chem 286(14):12554–12564. 10.1074/jbc.M110.182238 21321123 10.1074/jbc.M110.182238 PMC3069457

[CR101] Wegrzynowicz M, Holt HK, Friedman DB, Bowman AB (2012) Changes in the striatal proteome of YAC128Q mice exhibit gene-environment interactions between mutant huntingtin and manganese. J Proteome Res 11(2):1118–1132. 10.1021/pr200839d 22191580 10.1021/pr200839d PMC3319668

[CR102] Wiatr K, Szlachcic WJ, Trzeciak M, Figlerowicz M, Figiel M (2018) Huntington disease as a neurodevelopmental disorder and early signs of the disease in stem cells. Mol Neurobiol 55(4):3351–3371. 10.1007/s12035-017-0477-7 28497201 10.1007/s12035-017-0477-7 PMC5842500

[CR103] Wynford-Thomas R, Robertson NP (2017) The economic burden of chronic neurological disease. J Neurol 264(11):2345–2347. 10.1007/s00415-017-8632-7 29038885 10.1007/s00415-017-8632-7 PMC5686245

[CR104] Yang N, Higuchi O, Ohashi K, Nagata K, Wada A, Kangawa K, Nishida E, Mizuno K (1998) Cofilin phosphorylation by LIM-kinase 1 and its role in Rac-mediated actin reorganization. Nature 393(6687):809–812. 10.1038/31735 9655398 10.1038/31735

[CR105] Zhang J, Ooi J, Utami KH, Langley SR, Aning OA, Park DS, Renner M, Ma S, Cheok CF, Knoblich JA, Ginhoux F, Petretto E, Pouladi MA (2019) Expanded huntingtin CAG repeats disrupt the balance between neural progenitor expansion and differentiation in human cerebral organoids. bioRxiv:850586. 10.1101/850586

[CR106] Zhang X, Bearer EL, Boulat B, Hall FS, Uhl GR, Jacobs RE (2010) Altered neurocircuitry in the dopamine transporter knockout mouse brain. PLoS ONE 5(7):e11506. 10.1371/journal.pone.0011506 20634895 10.1371/journal.pone.0011506 PMC2901340

[CR107] Zhang Y, Li M, Drozda M, Chen M, Ren S, Mejia Sanchez RO, Leavitt BR, Cattaneo E, Ferrante RJ, Hayden MR, Friedlander RM (2003) Depletion of wild-type huntingtin in mouse models of neurologic diseases. J Neurochem 87(1):101–106. 10.1046/j.1471-4159.2003.01980.x 12969257 10.1046/j.1471-4159.2003.01980.x

[CR108] Zhao ZS, Manser E (2012) PAK family kinases: Physiological roles and regulation. Cell Logist 2(2):59–68. 10.4161/cl.21912 23162738 10.4161/cl.21912 PMC3490964

[CR109] Zielonka D, Marinus J, Roos RA, De Michele G, Di Donato S, Putter H, Marcinkowski J, Squitieri F, Bentivoglio AR, Landwehrmeyer GB (2013) The influence of gender on phenotype and disease progression in patients with Huntington’s disease. Parkinsonism Relat Disord 19(2):192–197. 10.1016/j.parkreldis.2012.09.012 23102616 10.1016/j.parkreldis.2012.09.012

