# Supplementary figures and images for: A Progressive Loss of phosphoSer138-Profilin Aligns with Symptomatic Course in the R6/2 Mouse Model of Huntington’s Disease: Possible Sex-Dependent Signaling
Source: Cell Mol Neurobiol. 2020 Oct 27;42(3):871–88. doi: 10.1007/s10571-020-00984-2 (PMC8891113; doi:10.1007/s10571-020-00984-2)

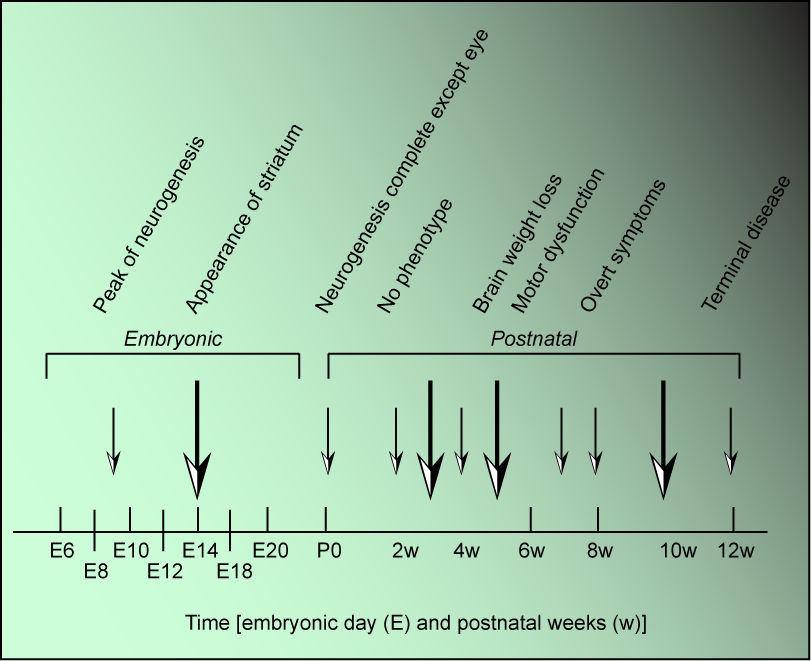

Supplement: Supplementary file 1 — Supplementary file1 Supplementary Figure 1: Schematic diagram showing the timeline of disease progression in R6/2 mouse brain [adapted from (Davies et al. 1997)]. The time is indicated in days for the embryonic (E) time points, ‘P0’ for ‘birth’, and weeks (w) for postnatal time points. The progression of phenotypes is indicated above the arrows. The large arrows indicate the time points selected for the current analysis. The small arrows indicate additional developmental time points significant in the disease progression. (TIF 1605 kb) [file 10571_2020_984_MOESM1_ESM.tif]

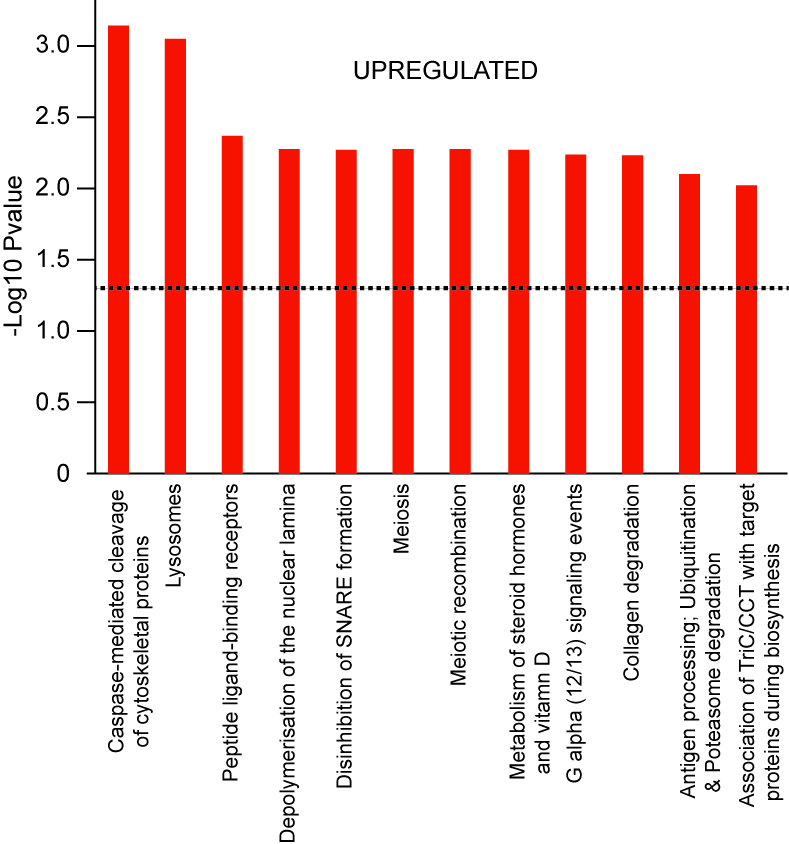

Supplement: Supplementary file 2 — Supplementary file2 Supplementary Figure 2: Bar graph generated by InnateDB using the output from PIIKA2 (details in Materials and Methods). Hyper-phosphorylated (positive) values from both sexes for all time points were analyzed and highly represented pathways are shown here (names across the x-axis). The y-axis represents the log p-value. Note, Innate DB summarizes the output from many online platforms such as KEGG, Reactome, PID NCI, PID Biocarta, NetPath, and INOH. As such, it is possible that the same pathways might be a ‘hit’ on several platforms and, for example, "Meiosis" had equal high scores across several platforms, as did “Meiotic recombination”. For sake of clarity, we simply listed any such multiple hits a single time in the graph. (TIF 3184 kb) [file 10571_2020_984_MOESM2_ESM.tif]

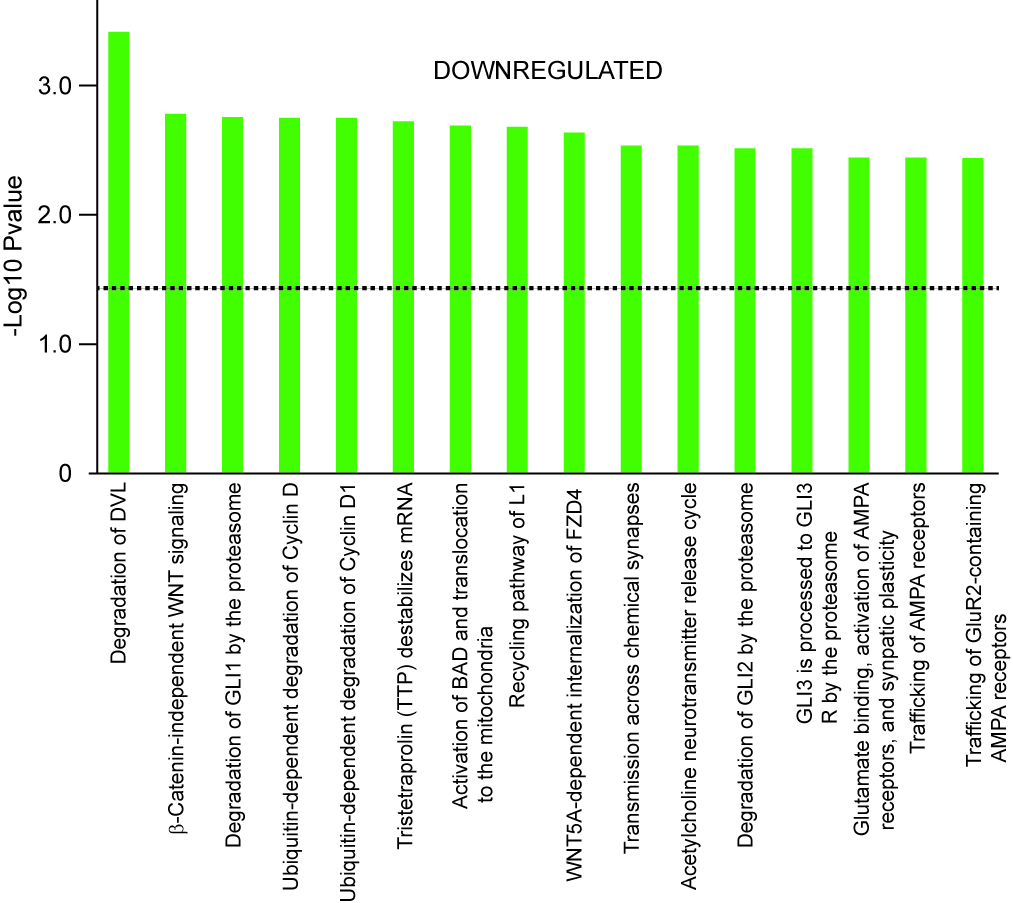

Supplement: Supplementary file 3 — Supplementary file3 Supplementary Figure 3: Bar graph generated by InnateDB using the output from PIIKA2 (details in Materials and Methods). Hypo-phosphorylated (negative) values from both sexes for all time points were analyzed and highly represented pathways are included here (names across x-axis). The y-axis represents the log p-value. (TIF 4156 kb) [file 10571_2020_984_MOESM3_ESM.tif]

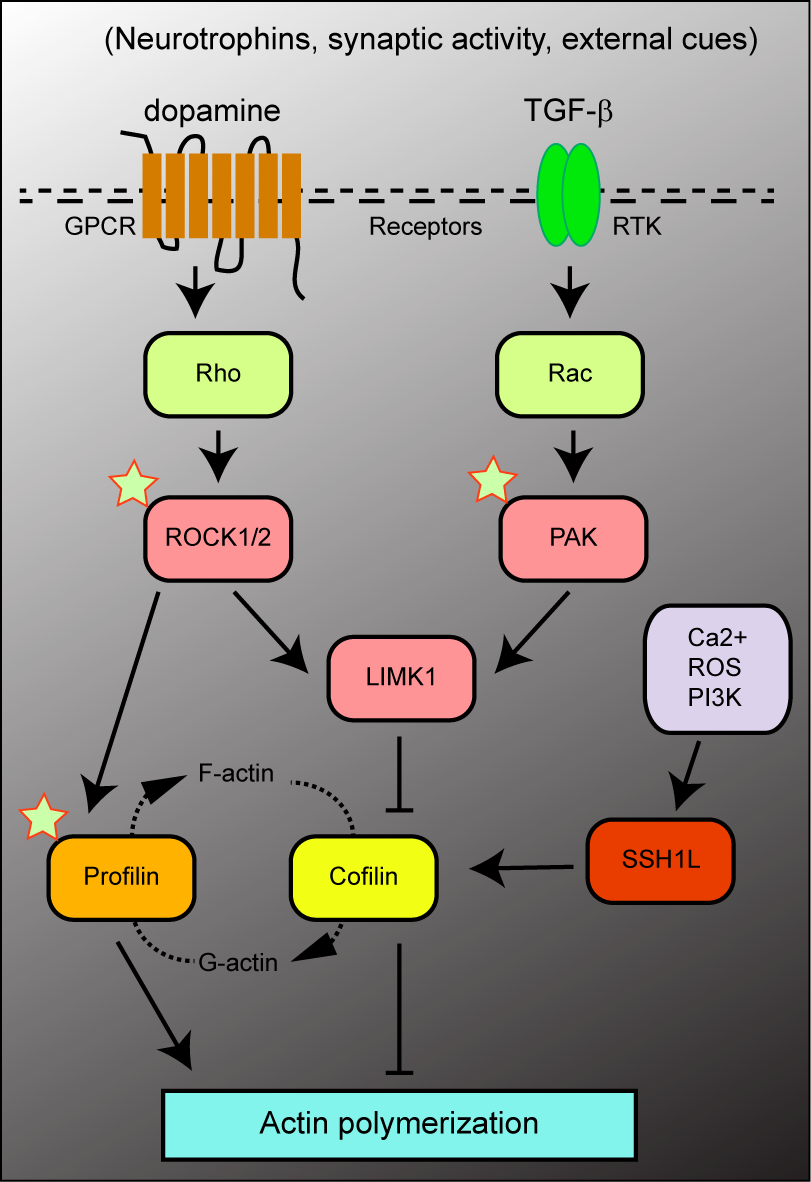

Supplement: Supplementary file 4 — Supplementary file4 Supplementary Figure 4: Molecular signaling involved in cytoskeletal organization: This illustration depicts the Rho-Rac signaling pathway, the key effectors, and the downstream targets that affect actin polymerization. The molecule labels represented in green and pink are kinases. External cues such as dopamine or transforming growth factor-β (TGF-β) can activate ROCK and PAK GTPases and trigger the phosphorylation and activation of LIMK, thus allowing the phosphorylation (and inactivation) of cofilin. Cofilin is dephosphorylated (reactivated) by the phosphatase SSH1L, which can be regulated by, for example, PI3K, ROS, and calcium (Ca2+). Profilin and cofilin are involved in maintaining the balance between filamentous (F) and globular (G) actin, both of which regulate actin polymerization. A star indicates a target in which a corresponding phosphopeptide was identified by kinome analysis. GPCR: G protein-coupled receptor; ROCK1/2: Rho-associated protein kinase 1/2; PAK: p21-activated kinase; LIMK1: Lim domain kinase 1; SSH1L: slingshot protein phosphatase 1; ROS: Reactive oxygen species; PI3K: Phosphoinositide 3´-kinase. (TIF 2845 kb) [file 10571_2020_984_MOESM4_ESM.tif]

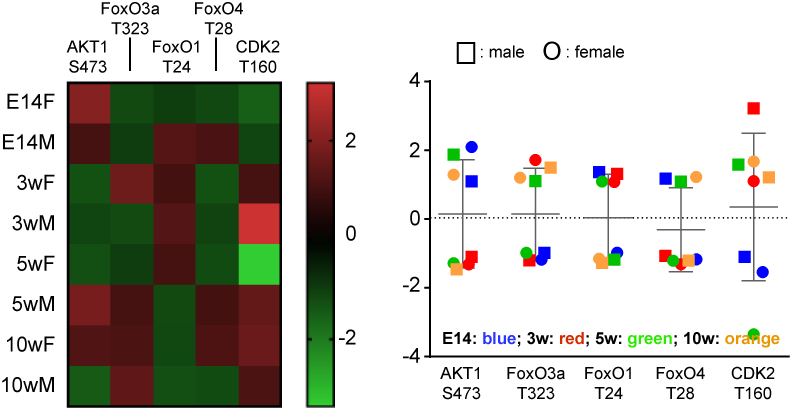

Supplement: Supplementary file 5 — Supplementary file9 Supplementary Figure 5: Changes in the Akt1/FoxO/CDK2 signaling pathway extracted from the kinome analysis of the R6/2 mice: (A) Fold-change heatmap across four time-points, e.g. E14, 3w, 5w, and 10w, in both sexes. The clustering was based on the time-points and the fold-change values. The color key represents positive values in red and negative values in green. The protein and the specific phosphosite are indicated at the top of each column. (B) Scatterplot of the fold-changes based on sex. Males are represented as squares and females as circles, with a different color assigned to each time-point, as indicated in the panel. (TIF 983 kb) [file 10571_2020_984_MOESM5_ESM.tif]

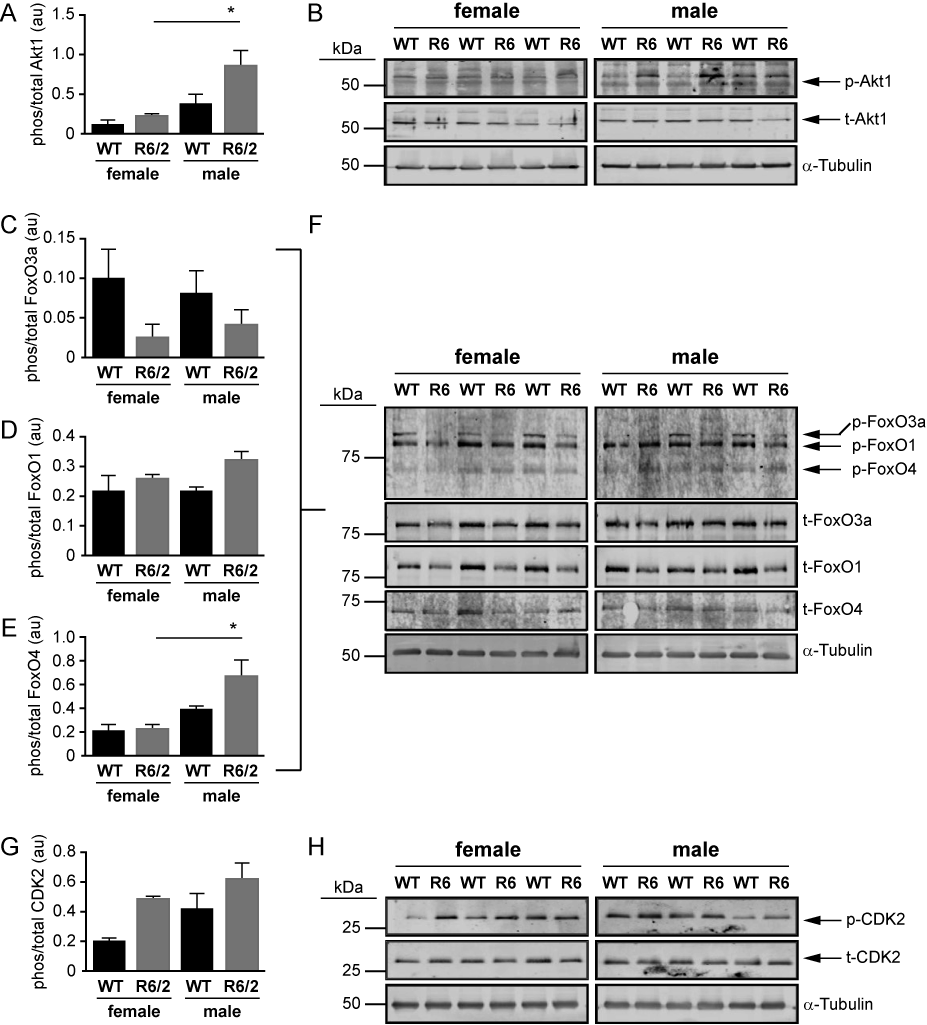

Supplement: Supplementary file 6 — Supplementary file11 Supplementary Figure 6: Western blots of the proteins involved in CDK2 signaling in wild type (WT) and R6/2 mice. Note, that due to limited tissue samples across the ages, we focused our Western blotting on the 10 week (10w) time-point. The average densitometry and corresponding Western blots of phosphorylated to total (A, B) Akt1, (C, D) FoxO proteins, and (E, F) CDK2 are shown. The anti-phospho-FoxO antibody detects FoxO1, FoxO3a, and FoxO4. The different isoforms are identified by their relative mobility on SDS-PAGE (kDa: kiloDalton). Each value was initially normalized to expression of α-Tubulin in the corresponding lane. Sex was considered as a variable in the statistical analyses using two-way ANOVA and post hoc multiple comparisons. The data are presented as mean ± sem (n=3). *: P < 0.05. (TIF 963 kb) [file 10571_2020_984_MOESM6_ESM.tif]

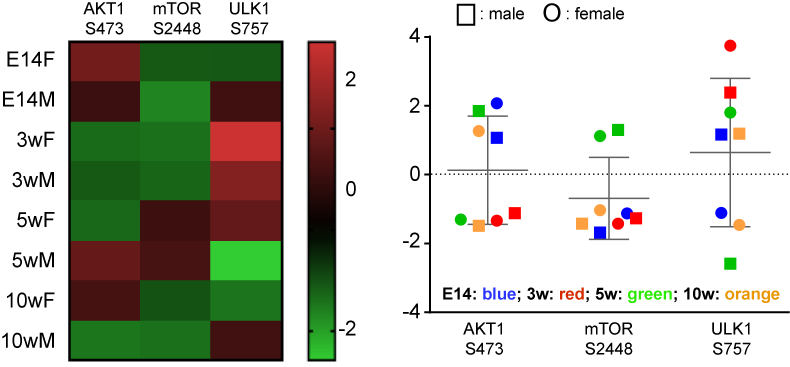

Supplement: Supplementary file 7 — Supplementary file12 Supplementary Figure 7: Changes in selected phosphorylated peptides in the Akt/mTOR/ULK1 cascade identified in the kinome analysis of the R6/2 mice: (A) Fold-change heatmap across four time-points, e.g. E14, 3w, 5w, and 10w, in both sexes. The clustering was based on the time-points and the fold-change values. The color key represents positive values in red and negative values in green. The names of the peptides and the phosphosite are indicated at the top of each column. (B) Scatterplot of the fold-changes based on sex. Males are represented as squares and females as circles, with a different color assigned to each time-point, as indicated in the panel. (TIF 872 kb) [file 10571_2020_984_MOESM7_ESM.tif]

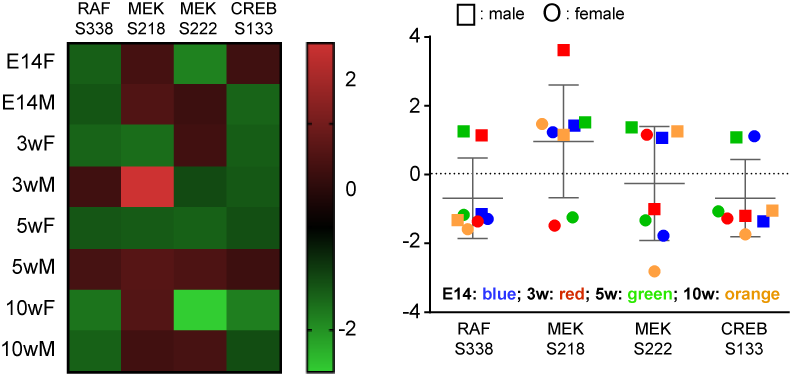

Supplement: Supplementary file 8 — Supplementary file13 Supplementary Figure 8: Changes in selected phosphorylated peptides in the RAF/MEK/CREB cascade identified in the kinome analysis of the R6/2 mice: (A) Fold-change heatmap across four time-points, e.g. E14, 3w, 5w, and 10w, in both sexes. The clustering was based on the time-points and the fold-change values. The color key represents positive values in red and negative values in green. The names of the peptides and the phosphosite are indicated at the top of each column. (B) Scatterplot of the fold-changes based on sex. Males are represented as squares and females as circles, with a different color assigned to each time-point, as indicated in the panel. (TIF 903 kb) [file 10571_2020_984_MOESM8_ESM.tif]
